# Maternal Taishan Panshi San supplementation is associated with improved foal growth performance, accompanied by alterations in gut microbiota and metabolic profiles

**DOI:** 10.3389/fmicb.2026.1787938

**Published:** 2026-04-20

**Authors:** Han Fu, Jianlong Li, Ronghao Miao, Zhanhai Mai, Yabin Lu, Ying Xiao, Ling Kuang, Qingyong Guo

**Affiliations:** 1 College of Veterinary Medicine, Xinjiang Agricultural University, Urumqi, Xinjiang, China; 2 Xinjiang Key Laboratory of New Drug Research and Development for Herbivores, Urumqi, Xinjiang, China

**Keywords:** blood hormones, foal, growth performance, short-chain fatty acids, Taishan Panshi San, Thoroughbred mares

## Abstract

**Objective:**

To investigate the effects of Taishan Panshi San (TSPSS) supplementation on foal growth and immunity.

**Methods:**

Twelve pregnant mares and their foals were divided into three groups: control (NC), TSPSS every other month (GY), and TSPSS monthly (MY) from gestation day 30. Maternal blood was sampled on days 65, 185, and 305 for hormone analysis. Foals were assessed for growth, cytokines, antioxidants, and immunoglobulins at birth, and onset of diarrhea (FQ), from 30 to 120 days after birth. Intestinal microbiota and metabolites were analyzed.

**Results:**

GY foals showed elevated levels of IL-6, IFN-γ, TNF-α, GH, IGF-I, insulin, IgA, IgG, IgM, SOD, and T-AOC (*p* < 0.05 or *p* < 0.01), with reduced MDA (*p* < 0.01). TSPSS upregulated placental nutrient transporters, vascular genes, and hormone receptors. GY foals exhibited increased abundance of *Synergistetes* spp. and decreased abundance of *Chlamydiae* spp. and *Phascolarctobacterium* spp., which correlated with enhanced bile acid synthesis. MY foals showed enrichment of *Odoribacter* spp. and *Akkermansia* spp. at FQ and *Ruminococcus* spp. at 90 d, which were linked to steroid and amino acid metabolism. Short-chain fatty acids (such as acetate, etc.) decreased at FQ/30 d, with group-specific correlations.

**Conclusion:**

Maternal TSPSS supplementation during pregnancy may modulate placental gene expression and improve gut microbiota and metabolism, thereby promoting the growth and development of foals.

## 1 Introduction

Xinjiang is a key province for the development of the horse industry in China. By the end of 2021, the total number of horses in the country was 3.725 million, with Xinjiang accounting for the largest share at 1.057 million (National Bureau of Statistics of China, 2022). To enhance the production performance of local horses in breeding, sports, dairy, and meat production, increasing numbers of high-quality breeding stock including Thoroughbreds have been imported from abroad (Xiaobin et al., 2019; Meng, 2013). However, due to changes in the climate and environment in China, the survival and adaptation of imported local breeding horses poses a significant challenge (Zhang Q. et al., 2023). Selective breeding is an important foundation for promoting the development of the horse industry, but foals are more sensitive to non-optimal environmental conditions, with increased susceptibility to disease, resulting in slow growth and development and decreased physical fitness (He, 2020). Pregnancy and postpartum periods are recognized as inducing remarkable physiological and metabolic adaptations in mammals. Despite the action of homeostatic mechanisms to maintain blood parameters within physiological levels, changes in metabolites and hormones occur as a result of increased metabolic demands in lactating animals. These changes are not necessarily indicative of disease but make animals physiologically unstable and more susceptible to a number of metabolic disorders at this stage than during other life periods, compromising productivity. Particularly, lactation is a demanding period for the animal, requiring a significant physiological effort to meet all its demands, while also placing increased pressure on farmers to provide adequate nutritional and management support (Bazzano et al., 2014; Arfuso et al., 2023; Fiore et al., 2017).

Previous studies have shown that the early, middle, and late stages of pregnancy in mares correspond to the first 4 months, months 5–8, and months 9–11 of gestation, respectively (Grossi et al., 2019). Fluctuations in estradiol (E2) and progesterone (P4) levels in mares during early pregnancy can adversely affect the normal development of the foal, and the mother's nutritional status can also directly influence the size and health of the foal at birth (Huntington et al., 2020). Thus, nutritional management during the pregnancy of pregnant mares is crucial to the growth and development of their offspring. To address this challenge, horse breeders often use pharmacoactive additives such as antimicrobials to improve the physical condition of mares during pregnancy, prevent disease, and enhance production efficiency (Colombo et al., 2022). However, the issue of bacterial antimicrobial resistance has become increasingly serious, posing risks to both animal and human health, including treatment failure, and has promoted nationwide ban on growth-promoting antibiotics as feed additives since 2020 (Ayalew et al., 2022). In recent years, the continued search for alternatives to conventional antibiotic drugs has led researchers to explore traditional Chinese medicine (TCM) as a source of safer, effective, natural compounds. TCM feed additives have a long history of safety and efficacy and have attracted considerable attention due to their variety of bioactive components, multiple mechanisms of activity, and favorable safety (Redoy et al., 2020). These additives can promote healthy development, improve production performance in animals, and provide additional health benefits such as immune enhancement and disease prevention (Cheng et al., 2020). Compared to synthetic drugs, TCM additives offer advantages in terms of significantly improving animal growth, reproduction, lactation, and immunity, while exhibiting limited side effects, low incidence of drug resistance, and no environmental residues (Yang, 2021).

Studies have shown that the TCM feed additive, Taishan Panshi San (TSPSS) can accelerate animal growth and development, enhance immunity, and improve digestion and absorption functions (Zou et al., 2021). This is largely attributed to its key ingredients, including Radix Rehmanniae Preparata (Zhang H. et al., 2023), Milkvetch Root (Alagawany et al., 2022), and Baical Skullcap Root (Hu et al., 2023), which promote fetal safety by enhancing antioxidant capacity and nourishing the blood. Specifically, the other TSPSS ingredients, such as *Radix Paeoniae Alba* (Sun et al., 2024) and *Largehead Atractylodes Rh* (Zhai et al., 2024) are reported to invigorate the spleen and stomach, eliminate the dampness, and promote fetal safety, while *Angelicae Sinensis Radix* (Zhang et al., 2018) increases the hemoglobin content and provide supplementary vitamins and trace elements. Thus, TSPSS improves the growth and development of both mares and their offspring. Consequently, dietary supplementation with TSPSS has been widely accepted as a recognized as an effective and scientifically sound feeding practice with far-reaching application potential in the field of animal husbandry (Mai et al., 2022).

The gut microbiota, as the “second brain” of the host, plays an indispensable role in the growth and development of animals (Aranda, 2021). The main fermentation products of intestinal microorganisms, short-chain fatty acids (SCFAs), not only provide energy for animals but also strengthen the intestinal barrier and support other beneficial physiological effects (Zhang L. Y. et al., 2021). To date, the mechanism underlying the effects of TSPSS on pregnant mares and their offspring has not been fully understood. Thus, this study, based on metabolomics and microbiomics techniques, will help elucidate the regulatory mechanism of TSPSS' action on pregnant mares and foals. Our data provide a scientific basis for the application of TSPSS in improving the efficiency of equine reproduction and ensuring the healthy growth of foals under equine reproduction management. We also demonstrated the clear advantages of TCM feed additives in horse feeding and management by integrating existing research findings on these additives with our results and providing practical recommendations.

## 2 Materials and methods

### 2.1 Experimental animals

Between April 2021 and September 2022, 12 pregnant local bred purebred English Thoroughbred mares (the ages of the selected mares ranged from 8 to 14 years, with a parity of 4–8, weighting 500 ± 50 kg), all reared at the Yili Horse Breeding Center, Zhaosu, China, and subjected to regular deworming, were used and their 12 foals aged 0–90 days were selected for experimentation at the Yili Horse Research and Breeding Center, Zhaosu County, Yili Kazakh Autonomous Prefecture, China. All experimental horses were clinically healthy. Pregnant mares were housed in individual stalls within a fully enclosed double-row stable. During daylight hours, the horses were turned out in a 50 m × 50 m outdoor exercise paddock featuring natural shade, returning to the stables at night. The experimental protocol was approved by the Animal Conservation and Ethics Committee of Xinjiang Agricultural University (License No. 2021076).

This study selected 12 pregnant mares that met the inclusion criteria and randomly assigned them to three groups using computer-generated random number tables: the control group (NC), the group receiving TSPSS supplementation every other month (GY), and the group receiving TSPSS supplementation monthly (MY), with four mares in each group. A double-blind approach was implemented throughout the experiment: during sample collection, personnel were aware only of the animal codes, not the group assignments; during laboratory analysis, all samples underwent anonymous coding, with analysts remaining entirely unaware of their groupings. Coding and group assignments were only reconciled after the completion of the experiment, effectively minimizing potential subjective bias.

### 2.2 Materials and instruments

Materials and equipment used in this study mainly include: equine-specific kits for estradiol (E_2_), progesterone (P_4_), interleukin-6 (IL-6), interleukin-10 (IL-10), interferon-γ (IFN-γ), and tumor necrosis factor-α (TNF-α; Jiangsu Meibiao Biotechnology Co., Ltd., Yan Cheng, China); kits for superoxide dismutase (SOD), malondialdehyde (MDA), and total antioxidant capacity (T-AOC; Suzhou Geruisi Biotechnology Co., Ltd., Su Zhou, China); kits for immunoglobulin A (IgA), immunoglobulin G (IgG), and immunoglobulin M (IgM), and the kits for growth hormone (GH), triiodothyronine (T3), tetraiodothyronine (T4), insulin-like growth factor-I (IGF-I), and insulin (INS; Beijing Huaying Biotechnology Research Institute Co., Ltd, Beijing, China); the BioTek Synergy HTX microplate reader (Beckman Coulter, Inc., United States); the automatic rapid sample grinding machine (The automatic rapid sample grinding machine, Shanghai, China); the constant temperature mixer (Eppendorf, Hamburg, Germany); the magnetic bead extractor (Thermo-Fisher Scientific, Massachusetts, United States); the MagPure stool DNA KF Kit B (Meiji Biotechnology Co., Ltd, Guangzhou, China); the Qubit™ dsDNA BR assay kit (Invitrogen Life Technologies, Massachusetts, United States); 2 × PhantaMax Master Mix (Vazyme Biotech Co., Ltd, Nanjing, China); the DNA sorting magnetic beads (BGI, Shenzhen, China); the MiSeq reagent kit v3 (600 cycle; Illumina, Inc., California, United States); the Quantus™ fluorometer (Promega, Wisconsin, United States); the Evo M-MLV reverse transcription PreMix Kit and SYBR green ProTaq HS PreMix qPCR Kit (Hunan Aikerui Bioengineering Co., Ltd, Changsha, China); the gas chromatograph (Agilent Technologies, Shanghai, China); the reagent N-butanol and acetic acid standards (Shuangshuang Chemical Co., Ltd, Yantai, China); the propionic acid, isobutyric acid, N-butyric acid, isovaleric acid, and N-pentanoic acid (Macklin Biochemical Technology Co., Ltd, Shanghai, China).

### 2.3 Experimental drugs

The TSPSS medication was prepared according to a previously published method (Mai et al., 2021). The dosage conversion from humans to animals was based on the recommended adult dosage of the component drugs and calculated using the following formula:


$$\begin{array}{l} \text{dose in mg}/\text{kg for animal B}=\text{W}\times \text{dose in mg}/\text{kg for animal A} \end{array}$$

where W is the body weight conversion coefficient, and the calculated dose for pregnant mares was 135 g per 400 kg body weight.

The ingredients of TSPSS were as follows: *Radix Codonopsis* (25 g), *Astragalus membranaceus* (15 g), *Atractylodes Macrocephala* (15 g), *Angelica Sinensis* (8 g), *Himalayan Teasel Root* (8 g), *Scutellaria Baicalensis* (3 g), *Rehmannia Glutinosa* (processed; 15 g), *Szechwan Lovage Rhizome* (6 g), *Paeoniae Radix Alba* (6 g), *Radix Glycyrrhizae Preparate* (5 g), *Villous Amomum* (6 g), *Citri Reticulataea Pericarpium* (6 g), *Ramulus Cinnamomic* 5 g, *Ginger* (6 g), and *Jujube* (6 g). These Chinese herbal medicines were purchased from Dongfang Kangtai Pharmacy in Zhaosu County, Yili, Xinjiang, China.

### 2.4 Feeding management

The mares were housed in individual stalls measuring 8 m × 5 m within a 50 m × 15 m barn overnight (20:00–8:00) and were allowed to graze and move freely in a 50 m × 50 m exercise yard during the daytime (8:00–20:00). The mares were fed a combination of roughage and concentrate at designated times, receiving five roughage meals (8:30, 11:30, 13:30, 16:30, 23:30) and two concentrated meals (6:00 and 20:00) per day. Feeding followed the principle of providing dry feed before wet and roughage before concentrate, with free access to water at all times. The foals accompanied the mares throughout the day and suckled freely. The composition and nutritional levels of the daily concentrate feed for the mares are detailed in Table 1.

**Table 1:** Ingredient and nutrient composition of the diet for pregnant mares.

Ingredient	Content (%)	Nutrient^b^	Content (%)
Corn	37.50	OM	90.64
Oat	20.00	CP	17.57
Wheat bran	15.00	DM	92.13
Barley	10.00	NDF	13.06
Soybean meal	15.00	ADF	28.35
Premix^a^	1.00	Ca^2+^	1.01
Limestone	1.00	AP	0.43
NaCl	0.50		
Total	100.00		

^a^The premix provided the following per kg of concentrate supplement: 480 IU of vitamin A, 816.32 mg of vitamin B_1_, 333.2 mg of vitamin B_2_, 48.96 mg of vitamin B_6_, 70.4 IU of vitamin D, 21,333.2 IU of vitamin E, and 20.46 mg of pantothenic acid, per kg of concentrate supplement, plus nicotinamide (484.85 mg), Cu (10.58 mg), Fe (5.56 mg), Mn (33.54 mg), Zn (30.92 mg), I (2.46 mg), Se (5.93 mg), and Co (1.11 mg);

^b^The nutritional levels of the concentrate were analytically determined.

### 2.5 Experimental design

**Figure 1:**
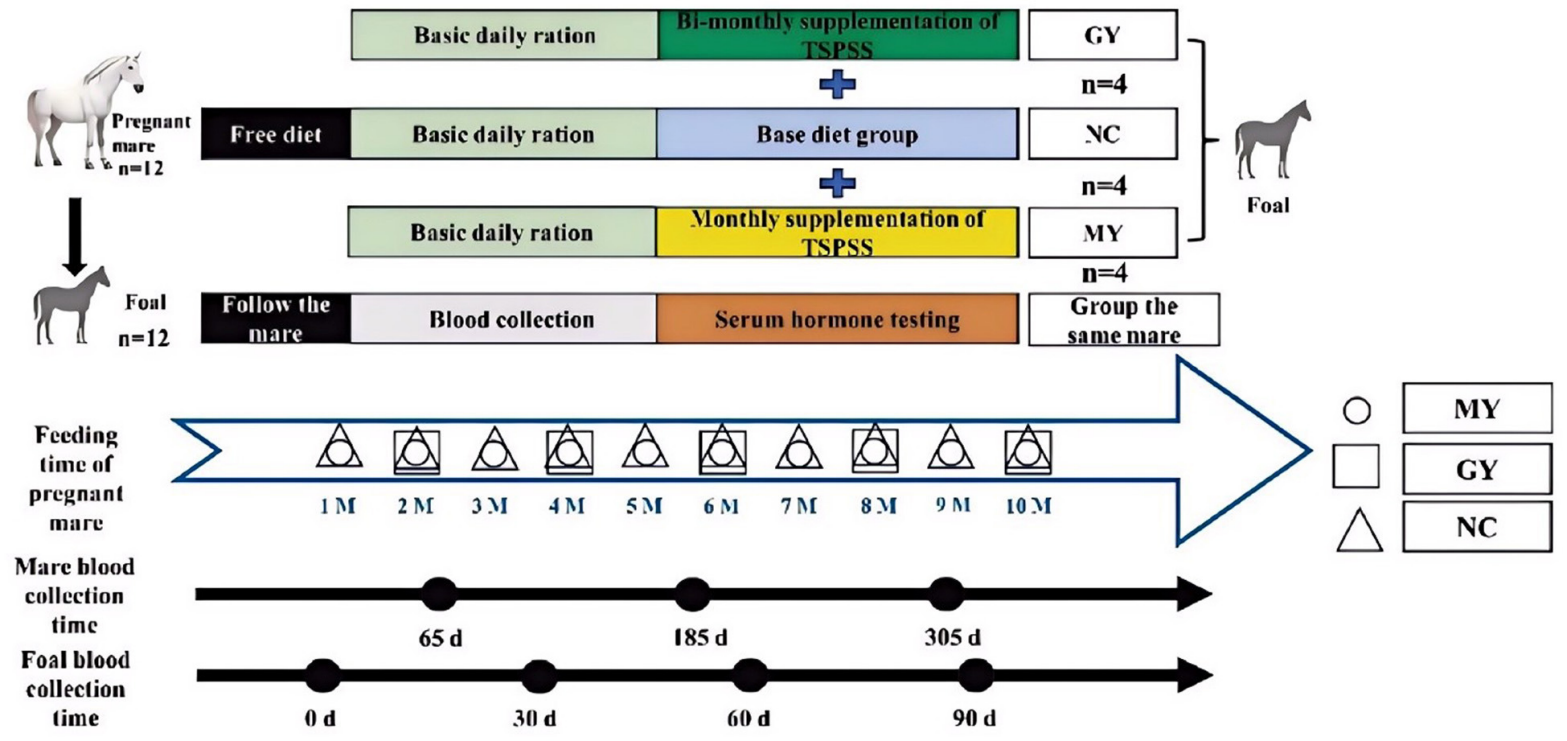
Flow chart of experimental design and sampling timeline.

Twelve local breeding purebred English Thoroughbred mares were randomly divided into three groups: control (MNC), bimonthly TSPSS supplementation in feed for five consecutive days (MGY), and monthly TSPSS supplementation in feed starting at 30 days of gestation for five consecutive days (MMY). All groups received the same basal ration composition and were managed under the same feeding and housing conditions. The foals were assigned into three similar groups: control (NC), bimonthly feeding (GY), and monthly feeding (MY), based on the groups of the mares from which they were born. Blood samples were collected from the mares on days 65, 185, and 305 of gestation, and blood samples were collected from the foals on day 0, at the first occurrence of diarrhea (FQ), and on days 30, 60, 90, and 120 after birth. The first episode of physiological diarrhea in newborn foals, typically occurring between 7 and 14 days of age, was defined as a critical window for intestinal microbiota colonization, gut barrier maturation, and immune function establishment. This process plays a key role in shaping the foals' intestinal microbial profile, short-chain fatty acid metabolism, and overall health. Therefore, the physiological characteristics of foals during this critical window can accurately reflect the regulatory effects of maternal TSPSS supplementation during pregnancy. At the same time, the foals were measured for body size and weight. Blood and fecal samples were collected between 10:00 am and 12:00 pm. All animals were handled by the same individual, and the sampling sequence was kept consistent. Blood was collected via jugular vein puncture using sterile needles and transferred into EDTA-containing tubes and standard vacuum tubes (Grans, Jixian County Medical Devices, Nanchang City, Jiangxi Province, China). Blood samples were transported immediately on ice to the laboratory, processed within 40 min, and stored at −20 °C. Fecal samples were collected using the rectal sampling method, flash frozen in liquid nitrogen, and stored at −80 °C until further analysis. The experimental design and sampling timeline are shown in Figure 1.

### 2.6 Administration method

The TSPSS formulation was crushed and homogeneously mixed into the feed concentrate. The pregnant mares in the MGY and MMY were fed the TCM additive TSPSS at 20:00 each day, following the protocol outlined in the experimental design.

### 2.7 Blood sample collection and processing

Blood samples were collected from pregnant mares on days 65, 185, and 305 of gestation following 12 h of supplementation of TSPSS. Fasting blood samples (5 mL) were collected from the foals in each group on days 0, 30, 60, and 90 after birth, allowed to clot in a 37 °C water bath for 10 min, and the serum was obtained by centrifugation at 3,000 × g for 10 min. The serum samples were aliquoted in equal volumes into 1.5 mL centrifuge tubes and stored at −20 °C until use.

### 2.8 Foal body size and weight measurements

Using the same sampling time points, body measurements including weight, height, oblique length, chest circumference, and cannon bone circumference were conducted on the foals at 0, 30, 60, and 90 days of age.

### 2.9 Determination of serum parameters

The concentrations of equine serum estradiol (E_2_, batch number: DF-50101A), progesterone (P_4_, batch number: DF-5017A), equine interleukin-6 (IL-6, batch number: MB-50005A), equine interleukin-10 (IL-10, batch number: MB-50009A), equine interferon-γ (IFN-γ, batch number: MB-2918A), and equine tumor necrosis factor-α (TNF-α, batch number: MB-50001A) were measured using a double-antibody one-step sandwich enzyme-linked immunosorbent assay (ELISA) kit according to the manufacturer's instructions (Jiangsu Meibiao Biotechnology Co., Ltd., Yan Cheng, China).

The contents of malondialdehyde (MDA, batch number: G0109W) and superoxide dismutase (SOD, batch number: G0101W) contents, and total antioxidant capacity (T-AOC, batch number: G0115W) were determined following the kit instructions (Suzhou Geruisi Biotechnology Co., Ltd., Suzhou, China), with absorbance were measured by microplate reader.

The ELISA kits achieved a minimum detectable concentration of 0.1 pg/mL (based on the average of six independent experiments), with an intra-assay coefficient of variation (CV) below 10% and an inter-assay CV below 15%.

Serum levels of immunoglobulin A (IgA, batch number: HY-N0048), immunoglobulin G (IgG, batch number: HY-N0050), immunoglobulin M (IgM, batch number: HY-N0049), growth hormone (GH, batch number: HY-C00018), triiodothyronine (T_3_, batch number: HY-A0001), thyroxine (T_4_, batch number: HY-A0002), insulin-like growth factor-I (IGF-I, batch number: HY-H0024), and insulin (INS, batch number: HY-D0001) were measured using a radioimmunoassay (RIA) method. The kits for these measurements were provided by Beijing Huaying Biotechnology Research Institute (Beijing, China).

### 2.10 RT-qPCR primer design and amplification

Real-time quantitative PCR (RT-qPCR) was employed to determine the gene expression levels in the placenta of newborn foals. The primers were designed based on the cDNA sequences of SLC2A1, SLC6A4, SLC7A1, SLC27A1, SLC28A1, SLC38A2, SLC38A4, CD36, VEGFA, NOS3, IGF-2, AKT, HTR2A-5, HTR7, DNMT3B, and the internal reference GAPDH for horse (*Equus caballus*). A panel of 15 target genes was selected based on their critical roles in placental function and fetal development. The focus was on three core functional modules: (1) placental nutrient transport, including genes that encode transporters for nutrients such as glucose (SLC2A1), amino acids (SLC7A1, SLC38A2, and SLC38A4), fatty acids (SLC27A1 and CD36), serotonin (SLC6A4) and nucleosides (SLC28A1). These genes are fundamental to the placental transfer of nutrients and directly influence fetal growth. (2) Placental angiogenesis and fetal growth regulation, including genes involved in vascular development (VEGFA and NOS3) and the insulin-like growth factor (IGF) signaling pathway (IGF2 and AKT), which are key determinants of placental perfusion and fetal growth. (3) Pregnancy maintenance and epigenetic regulation, including serotonin receptor genes (HTR2A-5 and HTR7), which are involved in uterine and placental function, as well as the DNA methyltransferase gene (DNMT3B). Primer design was conducted using Primer3 web version 4.1.0 (https://primer3.ut.ee/), with primer sequences obtained from the qPrimerDB database, followed by alignment using Primer-Blast. The primers were synthesized by Shanghai Sangon Biotech Co., Ltd. (Shnghai, China), and detailed primer information is provided in the Appendix.

The detailed amplification conditions were as follows: initial denaturation at 95 °C for 30 s, then 45 cycles of denaturation at 95 °C for 5 s, annealing at 60 °C for 30 s; melting curve at 95 °C for 10 s, 65 °C for 60 s, and 97 °C for 1 s; cooling to 37 °C for 30 s. The relative mRNA expression levels were expressed as 2^−ΔΔ*Ct*^, where ΔΔCt = [(Ct _targetgene_–Ct _GAPDH_)_testgroup_–(Ct _targetgene_–Ct _GAPDH_)_Controlgroup_].

### 2.11 Composition and relative abundance of gut microbiota

Total DNA was extracted from the rectal contents of foals to determine the microbial community, following the operating procedures provided by Shenzhen BGI Genomics Technology Co., Ltd. (BGI, Shenzhen, China). The quality of the extracted DNA was assessed by 1% agarose gel electrophoresis. The universal primers used for this amplification were 338F (ACTCCTACGGGAGGCAGCAG) and 806R (GGACTACHVGGGTWTCTAAT). The V3–V4 region of the bacterial 16S rRNA gene was amplified using a PCR thermocycler with the following program: initial denaturation at 98 °C for 1 min, followed by 30 cycles of denaturation at 98 °C for 10 s, annealing at 50 °C for 30 s, and extension at 72 °C for 60 s, ending with a final extension at 72 °C for 10 min. PCR was performed in a mixture containing 4 μL of 5× FastPfu buffer, 2 μL of 2.5 mM dNTPs, 0.8 μL of forward primer (5 μM) and reverse primer (5 μM), 0.4 μL of FastPfu polymerase, and 30 ng of template DNA. The PCR products were separated on a 2% agarose gel and purified using the Qubit™ dsDNA BR assay kit (Invitrogen Life Technologies, Waltham, MA, USA). The concentration of the purified PCR products was determined using a Quantus™ fluorometer (Promega, Madison, WI, USA).

The sequencing library was prepared using the TruSeq Nano DNA LT Library Prep Kit for the Illumina platform, following the standard procedures recommended by BGI. The paired-end sequence reads were merged using FLASH version 1.2.11 to generate contigs. Raw reads were optimized using QIIME version 1.9.1. The merged Tags underwent OTU clustering analysis using USEARCH software (v 7.0.1090).

### 2.12 Measurement of SCFAs

Fecal samples (0.8 g) were thoroughly mixed with 0.8 mL of ultrapure water, allowed to stand at room temperature for 20 min, then centrifuged at 15,000 × g for 15 min at 4 °C. The supernatants were transferred to clean 2 mL Eppendorf tubes, mixed with another 0.8 mL of ultrapure water, and the above steps were repeated. The two supernatants were combined, mixed thoroughly, and centrifuged again. To 990 μL of supernatant, 10 μL of N-butanol was added, mixed thoroughly, and filtered through a 0.22 μm filter membrane. A 1-μL aliquot of the filtrate was injected into a gas chromatograph (GC) for identification and quantification of SCFAs.

### 2.13 Data processing

All statistical analyses were performed using IBM SPSS Statistics 28.0 software (IBM Corporation, Armonk, NY, USA). A generalized linear model (GLM) analyses were conducted on baseline and post-treatment data for mares and foals, as well as other parameters (including plasma and serum). The dependent variables comprised measured indicators, with time and treatment group designated as fixed factors in order to examine the time × treatment interaction effects. Between-group effects reflected the primary influence of the three treatment groups (MNC, MMY and MGY), while within-group effects indicated the primary influence of gestational sampling time points. Interaction effects embodied the combined influence of treatment group and time point. A significant interaction indicated substantial biological relevance. Residual probability plots confirmed the normal distribution of data. Multiple comparisons between groups were adjusted using Bonferroni correction to control for Type I errors. Dunnett's test was used to assess differences relative to the control across all time points, and Tukey's test was applied to identify differences within the treatment-by-time interaction. Bar histogram and Spearman correlation analyses were performed to determine the associations between measured indicators and facal microbiota, with figures generated using GraphPad Prism software (version 10.1.2, GraphPad Software, San Diego, CA, USA). Results are expressed as mean ± standard error of the mean (SEM). Data are presented as least squares mean (LSM) ± standard error of the mean (SEM). Significant differences within groups are denoted as ^*^p < 0.05 or ^**^p < 0.01. Significant differences between groups are indicated as ^v−*z*^*p* < 0.05 or ^V−*Z*^*p* < 0.01.

## 3 Results

### 3.1 Ingredients of the Chinese medicine feed additive TSPSS

**Figure 2:**
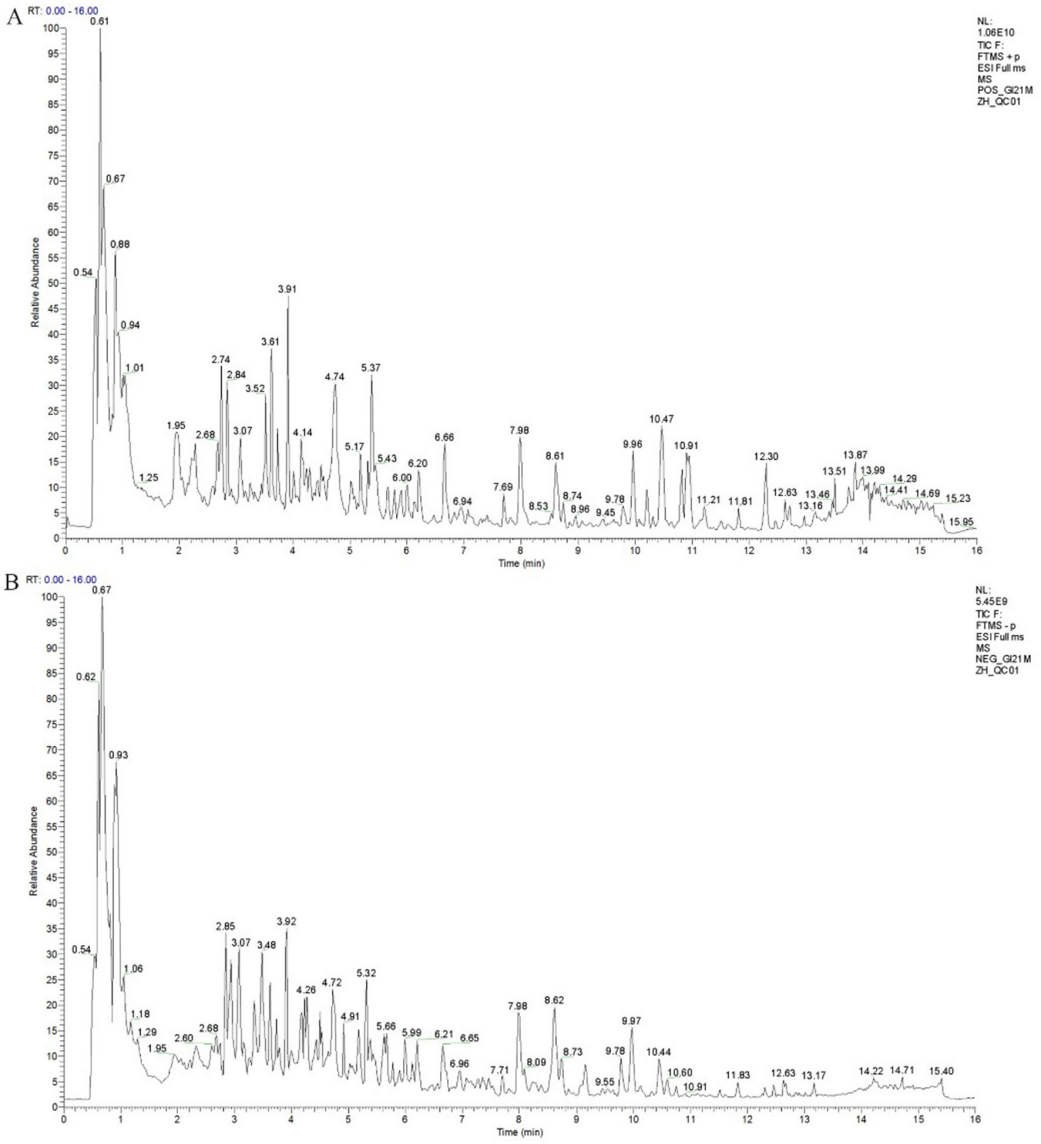
Total ion current diagram of the quality control sample. **(A)** C18 ESI (+); **(B)** C18 ESI (–).

**Figure 3:**
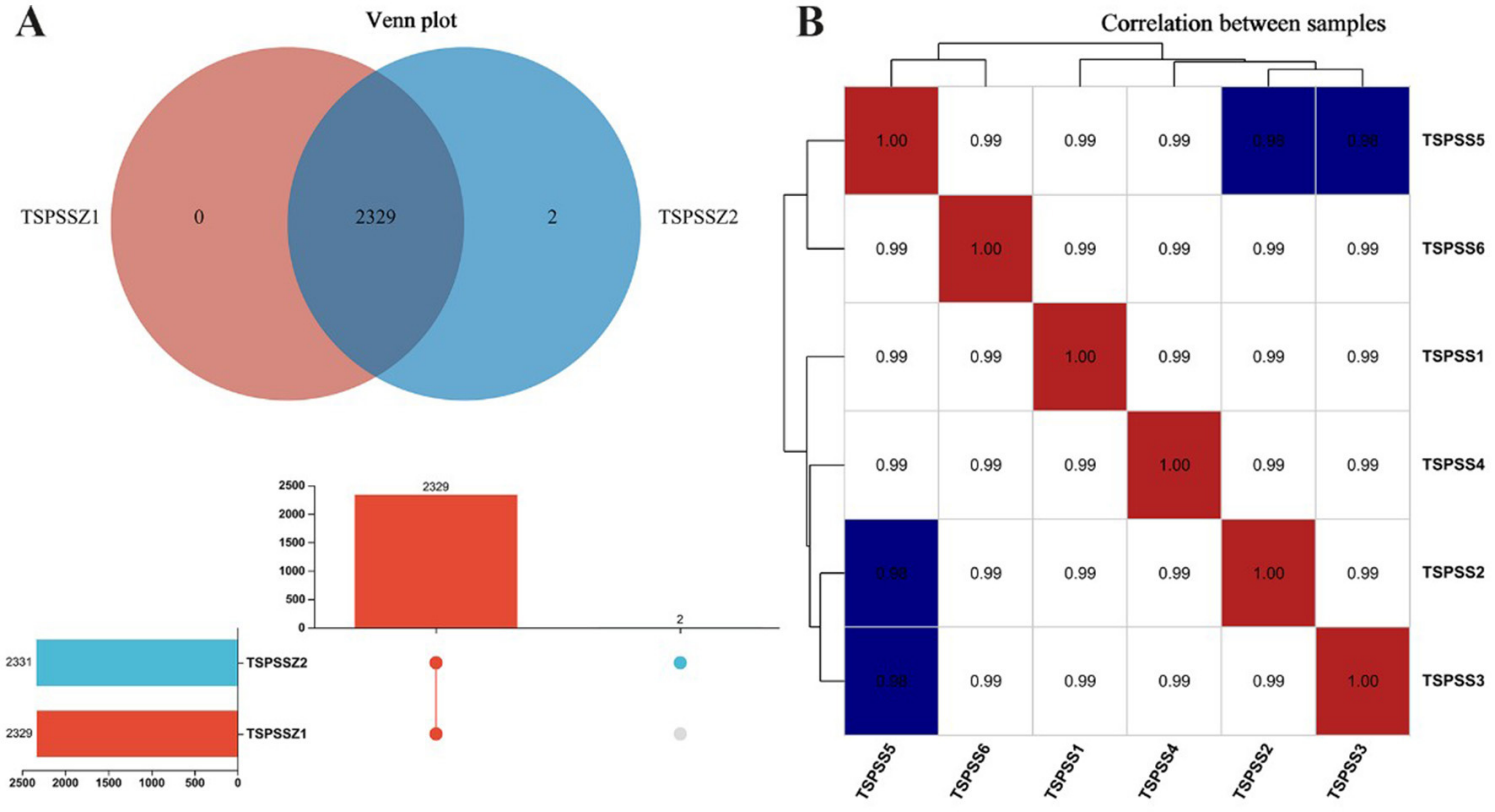
Similarity and correlation analysis results of metabolic composition of TSPSS samples. **(A)** Similarity of metabolic composition and abundance; **(B)** Sample repeatability.

During the chromatographic separation of TSPSS samples, the various components of the effluent were continuously sent to the mass spectrometer. The mass spectrometer performed sequential scans to collect data, with each scan generating a mass spectrum. The intensities of all ions in each mass spectrum were summed to obtain a total ion current intensity value. The total ion chromatograms of the QC samples showed clear peak shapes and relatively consistent peak distributions in both the positive and negative ion modes (Figure 2). To eliminate or reduce errors in the experimental and analytical sessions, the raw data were preprocessed by filtering, applying complementary values, normalization, and logarithmic transformation. Compound peaks and quantities were obtained after qualitative and quantitative analysis, and the results are shown in Table 2. After data preprocessing, the number of peaks in positive ion mode (Pos) was 1,135, and the number in negative ion mode (Neg) was 1,193. By quantitatively comparing the correlation data between the samples, the degree of variability in the metabolite compositions and abundances could be measured. A correlation value closer to 1 indicates a higher degree of similarity in metabolite composition and abundance across the samples. The results showed that all six drugs in both sets of TSPSS met the expected criteria in terms of reproducibility (Figure 3). The metabolites were screened based on a fragmentation score/theoretical fragmentation score ≥ 60. The major compounds in TSPSS were identified by LC-MS/MS, and the respective retention times (RT), m/z values, molecular formulae, errors (ppm), and MS/MS cleavage modes of the identified compounds were characterized and confirmed. The metabolites were screened, compared with the single drug components of TSPSS in the herb database, and the unidentified substances were removed. A total of 104 compounds were obtained, of which 54 were detected in positive ion mode and 50 in negative ion mode. A total of 104 compounds were identified (Supplementary Tables S1, S2), including 44 flavonoids, 18 terpenoids, 12 polyphenols and their derivatives, 9 organic acids and their derivatives, and 21 other compounds.

**Table 2:** Statistical summary of metabolites by ion mode.

Ion mode	Effective peak (origin)	Identified metabolites (origin)	Proportion (%)
Pos	12,107	1,135	9.37
Neg	15,610	1,193	7.64

### 3.2 Effect of TSPSS on E2 and P4 levels in pregnant mares at various stages of pregnancy

**Figure 4:**
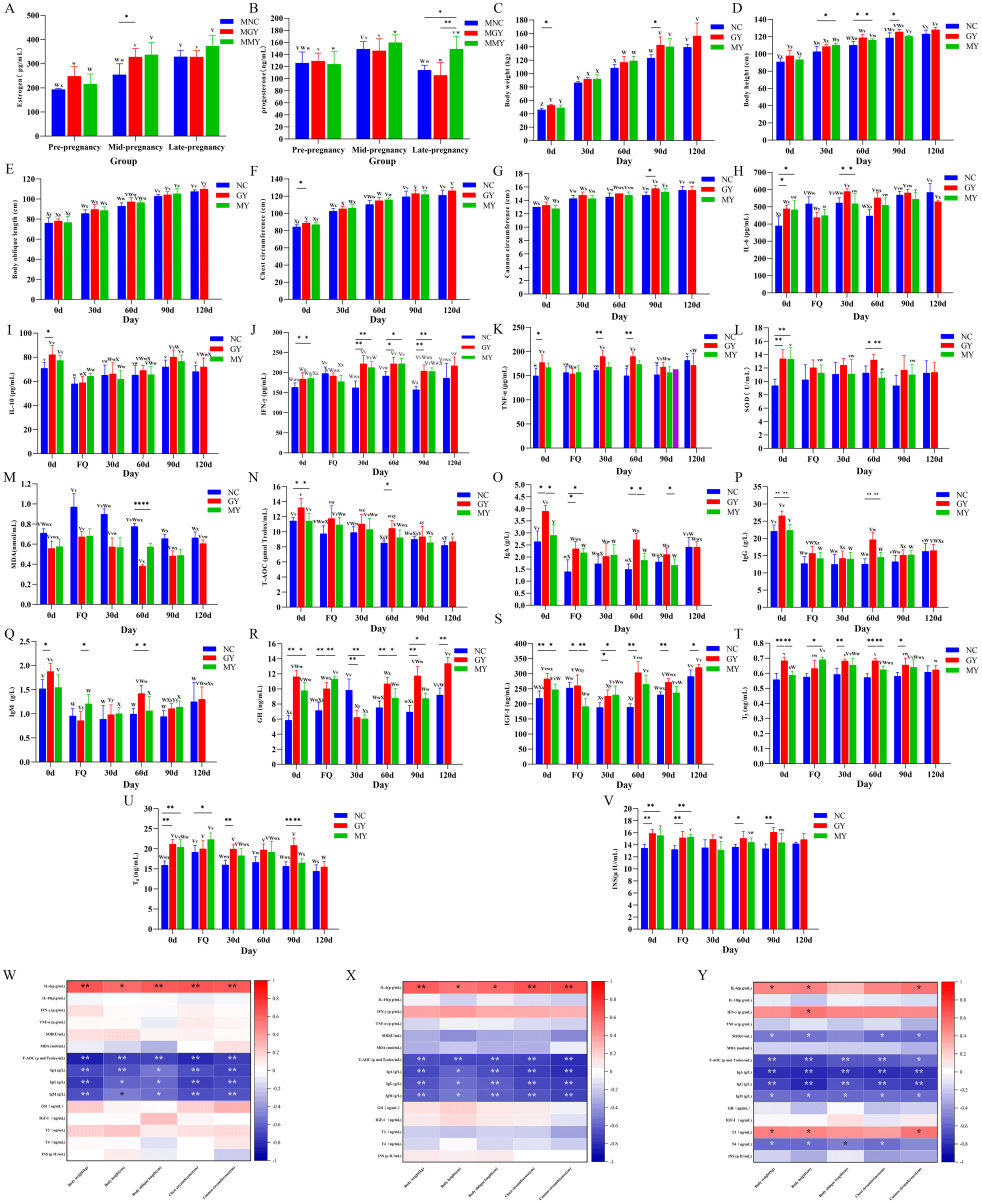
Detection of growth and development changes and serological indexes and correlation analysis of hormone levels and growth performance in foals. **(A, B)** Changes in body size and weight of foals from 0 to 90 days in each group. **(C)** Body weight (kg); **(D)** Body height (cm); **(E)** Oblique length (cm); **(F)** Chest circumference (cm); **(G)** Cannon circumference (cm). Data are presented as means ± standard deviation (*n* = 4). Changes in inflammatory factor levels in the serum of foals from 0 to 120 d in each group: **(H)** IL-6 concentration (pg/mL); **(I)** IL-10 concentration (pg/mL); **(J)** IFN-γ concentration (pg/mL); **(K)** TNF-α concentration (pg/mL). Data are presented as mean ± standard deviation (*n* = 4). Changes in antioxidant content and immunoglobulin levels in the serum of foals from 0 to 120 d in each group: **(L)** SOD concentration (U/mL); **(M)** MDA concentration (nmol/mL); **(N)** T-AOC (μmol Trolox/mL). Data are means ± standard deviation (*n* = 4). **(O)** IgA concentration (g/L); **(P)** IgG concentration (g/L); **(Q)** IgM concentration (g/L) Changes in hormone levels in the serum of foals from 0 to 90 d in each group: **(R)** GH content (ng/mL); **(S)** IGF-I content (ng/mL); **(T)** T3 content (ng/mL); **(U)** T4 content (ng/mL); **(V)** Ins content (μIU/mL). Data are means ± standard deviation (*n* = 4): Correlation between serum hormone indices and growth performance of mares on their foals supplemented with TSPSS: **(W)** Correlation of foals in NC group; **(X)** Correlation of foals in GY group; **(Y)** Correlation of foals in MY group. Significant differences within groups (*p < 0.05, **p < 0.01). Significant differences between groups (^v − z^*p* < 0.05, ^V − Z^*p* < 0.01).

Significant effects were observed under the main effects of developmental stage and grouping, as well as under the interaction between developmental stage and grouping, on levels of E2 and P4 in pregnant mares (Supplementary Table S3). E_2_ is one of the essential hormones for maintaining pregnancy, and its level in pregnant mares in all groups exhibited an increasing trend. Compared with the MNC group, the E_2_ levels in the MGY and MMY groups were significantly elevated during early and mid-gestation. Furthermore, E2 levels were significantly higher in the MGY group than in the MNC group during mid-pregnancy (*p* < 0.05). However, toward the late gestation period, only the mares in the MMY group showed a trend of increased E_2_ levels, although these differences were not statistically significant (*p* > 0.05; Figure 4A). This finding indicated that supplementation of TSPSS did not have a significant influence on E_2_ levels in pregnant mares. In contrast, compared with the MGY group, a significant increase in P_4_ levels was observed in the mares from the MMY group during late gestation (*p* < 0.05; Figure 4B). No significant changes in E_2_ or P_4_ levels were observed among the other groups or stages (*p* > 0.05).

### 3.3 Effect of TSPSS on body measurements and weight changes in foals aged 0–120 days

Significant effects were observed for the main effects of developmental stage and grouping, as well as for their interaction, on body measurements and weight changes of the foals (Supplementary Table S4). The results show a progressive increase in the body measurements and weights of the foals in all groups from birth to 120 days (Figure 4). Notably, compared with the NC group, the GY group exhibited significant increases in weight and chest girth at 0 and 90 days (*p* < 0.05) and a significant increase in body height at 30, 60, and 90 days (*p* < 0.05; Figures 4C–F).

### 3.4 Effect of TSPSS on serum inflammatory cytokine levels in foals aged 0–120 days

A significant effect was observed on the serum inflammatory cytokine levels of the foals, under the main effects of developmental stage and grouping, as well as their interaction (Supplementary Table S5). Compared with the NC group, the IL-6 content in the GY and MY group was significantly higher at 0 and 90 days of age (Figure 4). At 30 and 60 days, the IFN-γ and TNF-α levels in the GY groups were significantly elevated (*p* < 0.01, *p* < 0.05), indicating that the active ingredients in TSPSS have an impact on the immune system of foals at a young age. By 60 days, the IFN-γ content in both the GY and MY groups was significantly increased (*p* < 0.05), the TNF-α level in these groups was also significantly elevated (*p* < 0.01, *p* < 0.05). At 0 and 30 days, and the IL-6 content in the GY group was significantly increased (*p* < 0.05). At 30, 60, and 90 days, the IFN-γ level in the GY and MY groups was extremely significantly increased (*p* < 0.01, *p* < 0.05). At 0 days, and the IL-10 content in the GY group was significantly increased (*p* < 0.05; Figures 4H–K).

### 3.5 Effect of TSPSS on serum antioxidant marker levels and serum immunological parameters in foals aged 0–120 days

A significant effect was observed on the serum antioxidant marker levels of the foals, under the main effects of developmental stage and grouping, as well as their interaction (Supplementary Table S5). The SOD activity in the GY and MY groups was significantly greater than that in the NC group at 0 days of age (*p* < 0.01; Figure 4L). Furthermore, the SOD activity in the GY group was significantly greater than those in the NC and MY groups (*p* < 0.05, *p* < 0.01).

By 60 days of age, the MDA content in the GY and MY groups was significantly lower than that in the NC group (*p* < 0.01; Figure 4M). In addition, the T-AOC in the GY group was also significantly higher at 0 and 60 days (*p* < 0.05; Figure 4N).

Compared to the NC group, the IgA, IgG and IgM levels in the foals in the GY group were significantly elevated at 0 days of age (*p* < 0.01, *p* < 0.05). At 60 days, there was an extremely significant increase in IgA, IgG and IgM levels (*p* < 0.01, *p* < 0.05; Figures 4O–Q). This finding suggests that the foals in GY group effectively increased their Ig levels to better adapt to the impact of the external environment on their bodies. However, there were no significant differences in the IgA, IgG, and IgM levels between the MY group and the NC groups during the 0–90 days period (*p* > 0.05). Additionally, at 0 day, the IgA and IgG levels in the GY group were significantly greater than those in the MY group (*p* < 0.05, *p* < 0.01). At 60 days, the IgA, IgG and IgM levels in the GY group were significantly greater than those in the NC group (*p* < 0.05, *p* < 0.01).

### 3.6 Effects of TSPSS on serum growth factor levels in foals aged 0–120 days

A significant effect was observed on serum growth factor levels of the foals, under the main effects of developmental stage and grouping, as well as their interaction (Supplementary Table S5). Compared to the NC group, the GY group exhibited varying degrees of changes in GH, IGF-I, T3, T4, and INS levels from 0 to 90 days of age (Figures 4R–V). Specifically, at 0 days, the GY group had significantly elevated levels of GH, IGF-I, T3, T4, and INS (*p* < 0.01). At 30 days, the GY group showed a significant decrease in GH levels (*p* < 0.01) but a significant increase in T3 and T4 levels (*p* < 0.01), indicating that the foals were undergoing rapid growth and development, with highly active metabolic activities and a greater demand for growth hormones. At 60 days, the GY group had significantly elevated levels of GH, IGF-I, and T3 (*p* < 0.01). By 90 days, the GY group displayed significantly elevated levels of GH, IGF-I, INS, and T4 (*p* < 0.01), as well as significant increases in T3 (*p* < 0.05). Compared to the MY group, the GY group exhibited varying degrees of changes in GH, T3, IGF-I and T4 levels at 0, 30, 60, and 90 days of age. At 0 and 90 days, the GY group had significantly greater GH levels than the MY group (*p* < 0.05), significantly T3 levels at 60 days, and significantly greater T4 levels at 90 days compared with the MY group (*p* < 0.01).

### 3.7 Correlation between serum hormone levels and growth performance in foals aged 0–120 days under different supplementary feeding treatments

The results for the NC group (Figure 4W) indicate a strong positive correlation between IL-6 and body weight, body oblique length, chest circumference, and cannon bone circumference during suckling (*p* < 0.01), as well as a significant positive correlation with body height (*p* < 0.05). In contrast, in the NC group, T-AOC exhibited a significant negative correlation with body weight, body height, body oblique length, chest circumference, and cannon circumference (*p* < 0.01). IgA was significantly negatively correlated with body weight, body height, chest circumference, and cannon circumference (*p* < 0.01), while significantly negatively correlated with body oblique length (*p* < 0.05). IgG and IgM both displayed significant negative correlations with body weight, chest circumference, and cannon circumference (*p* < 0.01), as well as significant negative correlations with body height and body oblique length (*p* < 0.05). In the GY group (Figure 4X), IL-6 exhibited significant positive correlations with body weight, chest circumference, and cannon circumference during suckling (*p* < 0.01), as well as significant positive correlations with body height and body oblique length (*p* < 0.05). IgA, IgG, and IgM showed significant negative correlations with body weight, body oblique length, chest circumference, and cannon circumference (*p* < 0.01), as well as significant negative correlations with body height (*p* < 0.05). In the MY group (Figure 4Y), T3 displayed significant positive correlations with body weight, body height, and cannon circumference during suckling (*p* < 0.05). IFN-γ showed a significant positive correlation with body height (*p* < 0.05), indicating that the active components in TSPSS promote growth and development in suckling foals, accelerating muscle tissue growth and improving body height. SOD exhibited significant negative correlations with body weight, body height, chest circumference, and cannon circumference (*p* < 0.05). T-AOC, IgA, and IgG displayed significant negative correlations with body weight, body height, body oblique length, and chest circumference (*p* < 0.01), and T-AOC also showed a significant negative correlation with cannon circumference (*p* < 0.05). IgA and IgG exhibited significant negative correlations with cannon circumference (*p* < 0.01). IgM and T4 were significantly negatively correlated with body weight, body height, body oblique length, and chest circumference, and IgM was also significantly negatively correlated with cannon circumference (*p* < 0.05).

### 3.8 Effect of TSPSS on placental mRNA expression

**Figure 5:**
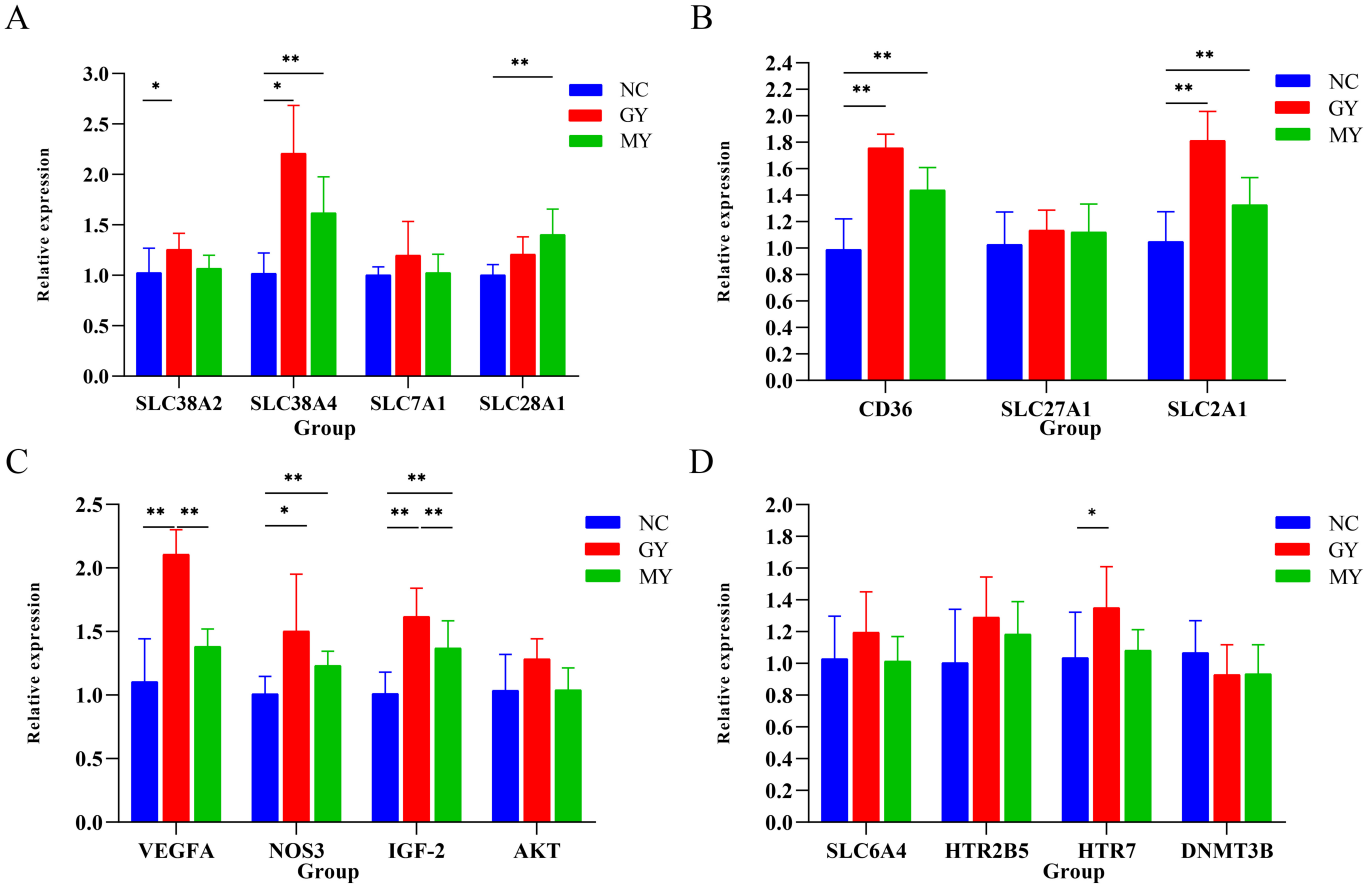
Relative expression levels of placental genes measured by RT-qPCR. **(A)** Amino acid transporter genes; **(B)** Fatty acid and glucose transporter genes; **(C)** Genes related to placental development; **(D)** Genes related to placental serotonin. Significant differences within groups: *p < 0.05, **p < 0.01.

Compared with the NC group, the GY group showed significant upregulation of the *SLC38A2, SLC38A4, NOS3*, and *HTR7* genes (*p* < 0.05) and highly significant upregulation of the *CD36, SLC2A1, VEGFA*, and *IGF-2* genes (*p* < 0.01). In the MY group, significant upregulation was observed for the *SLC2A1* gene (*p* < 0.05), while highly significant upregulation was observed for the *SLC38A4, SLC38A1, CD36, NOS3*, and *IGF-2* genes (*p* < 0.01). Furthermore, the expression levels of the IGF-2 and VEGFA genes were significantly higher in the GY group than in the MY group (*p* < 0.05 and *p* < 0.01, respectively; Figure 5).

### 3.9 Quality inspection of metagenomic DNA of foal intestinal microbiota treated with TSPSS

**Figure 6:**
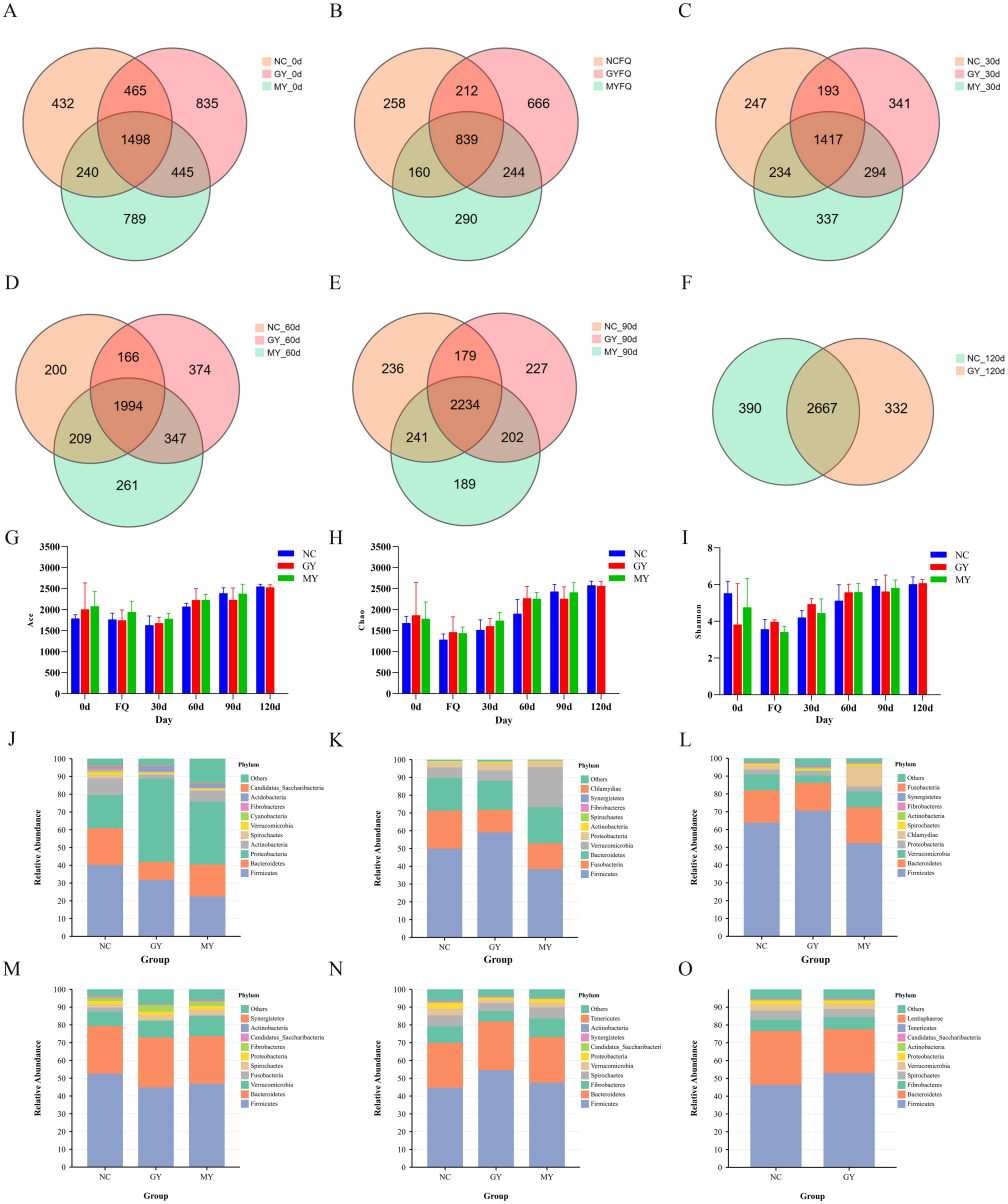
Venn diagram and α-diversity of foal intestinal microbiota. **(A)** Day 0; **(B)** first occurrence of diarrheas; **(C)** day 30; **(D)** day 60; **(E)** day 90; **(F)** day 120; **(G)** Chao index; **(H)** ACE index; **(I)** Shannon index. Relative abundance of foal intestinal microbiota at the phylum level. **(J)** Day 0; **(K)** at first occurrence of diarrhea; **(L)** day 30; **(M)** day 60; **(N)** day 90; **(O)** day 120.

A total of 8,850, 4,963, 6,518, 8,261, and 8,598 OTUs were detected across the foal groups from day 0 to day 90, respectively, with 1,498, 839, 1,417, 1,994, and 2,234 shared OTUs among the groups at each corresponding time point (Figures 6A–F). On day 120 after birth, a total of 6,056 OTUs were detected in the NC and GY groups, with 2,667 shared OTUs between the two groups. As depicted in Figures 6G–I, there were no significant differences in α-diversity among the foal groups (*p* > 0.05).

### 3.10 Changes in relative abundance of intestinal microbiota at the phylum level in foals

Gut microbiota analysis at the phylum level revealed that the six most dominant phyla in the intestinal microbiota of foals from day 0 to day 120 were Firmicutes, Bacteroidetes, Proteobacteria, Actinobacteria, Spirochaetes, and Verrucomicrobia (Figures 6J–O). Among them, Firmicutes was the dominant phylum, accounting for 22% to 70% of the total relative abundance. The top ten phyla were selected for significance testing. Compared with the NC group, the abundance of Chlamydiae in the GY group showed a significant decrease on day 30 after birth, while Synergistetes showed a significant increase (*p* < 0.05; Table 3).

**Table 3:** Relative abundance of intestinal microbiota at the phylum level in 30-day-old foals.

Item	NC	GY	MY	*p* value
Firmicutes	63.83 ± 18.88	70.49 ± 5.90	52.26 ± 9.19	0.246
Bacteroidetes	18.22 ± 16.82	15.71 ± 10.60	20.30 ± 4.30	0.595
Verrucomicrobia	8.84 ± 11.47	4.11 ± 4.95	8.71 ± 6.04	0.437
Proteobacteria	2.77 ± 2.85	2.71 ± 1.78	2.86 ± 2.34	0.981
Chlamydiae	2.35 ± 2.29	0.11 ± 0.04^*^	11.52 ± 11.17	0.015
Spirochaetes	0.89 ± 1.24	1.05 ± 1.10	1.03 ± 1.31	0.874
Actinobacteria	0.32 ± 0.12	0.50 ± 0.23	0.21 ± 0.02	0.050
Fibrobacteres	0.32 ± 0.41	0.35 ± 0.53	0.46 ± 0.71	0.779
Synergistetes	0.24 ± 0.19	0.91 ± 0.77^*^	0.10 ± 0.02	0.044
Fusobacteria	0.21 ± 0.19	0.13 ± 0.07	0.10 ± 0.07	0.618

Phyla marked with asterisk(s) indicate differences compared with the NC group:

^*^p < 0.05.

### 3.11 Changes in β-diversity of the equine gut microbiota

**Figure 7:**
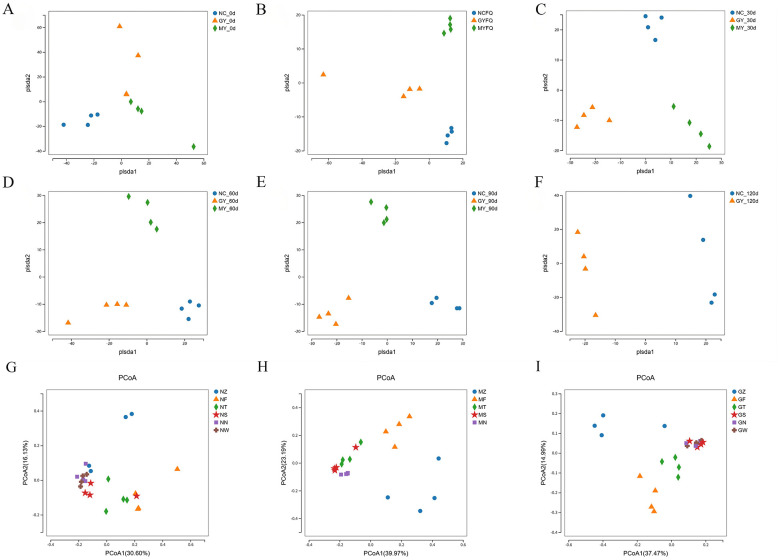
β-Diversity plot of the intestinal microbiota of foals. **(A)** Foals 0 days old; **(B)** Foals with diarrhea; **(C)** Foals 30 days old; **(D)** Foals 60 days old; **(E)** Foals 90 days old; **(F)** Foals 120 days old. **(G)** PCoA at various time points for the NC group; **(H)** PCoA at various time points for the MY group; **(I)** PCoA at various time points for the GY group.

**β**-diversity analysis was used to compare microbial community composition across different samples. PLS-DA analysis revealed that maternal TSPSS supplementation administered either every other month or monthly during gestation differentially affected the gut microbiota of foals from birth to 120 days of age. As demonstrated in Figures 7A–F, the intestinal microbiota of foals from different groups exhibited distinct OTU clustering patterns. As illustrated in Figures 7G–I, there were substantial variations in the composition of the gut microbial community among the NC, MY, and GY groups at six pivotal time points (day 0, FQ, days 30, 60, 90, and 120; *p* < 0.05). On days 30, 60, 90, and 120, the corresponding R^2^ values were 0.45, 0.56, and 0.65 for the NC, GY, and MY groups, respectively.

### 3.12 Cluster analysis of species abundance in foals at the genus level

**Figure 8:**
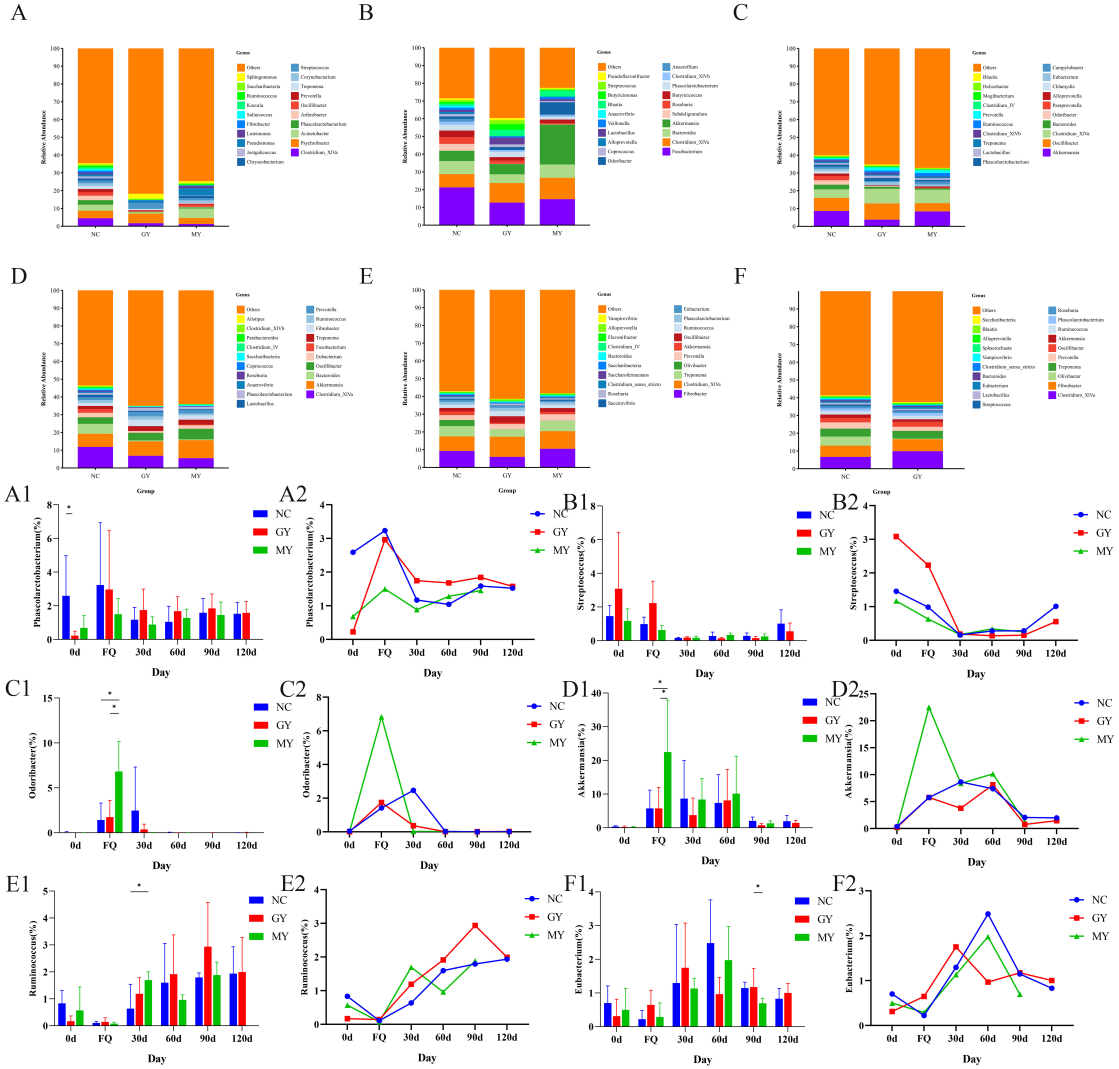
Effect of TSPSS treatment on the genus-level intestinal microbiota in foals (top 20). **(A)** Day 0 (birth); **(B)** first occurrence of diarrhea; **(C)** day 30; **(D)** day 60; **(E)** day 90; **(F)** day 120. **(A1, A2)** Relative abundance of *Phascolarctobacterium* spp.; **(B1, B2)** Relative abundance of *Streptococcus* spp.; **(C1, C2)** Relative abundance of *Odoribacter* spp.; **(D1, D2)** Relative abundance of *Akkermansia* spp.; **(E1, E2)** Relative abundance of *Ruminococcus* spp.; **(F1, F2)** Relative abundance of *Eubacterium* spp. Significant differences within groups: *p < 0.05.

Gut microbiota analysis at the genus level revealed dynamic shifts in the dominant genera in foals from day 0 to day 120 after birth (Figures 8A–F).

On day 0 (birth), the main microbiota consisted of *Clostridium_XlVa* spp., *Psychrobacter* spp., *Acinetobacter* spp., *Phascolarctobacterium* spp., *Arthrobacter* spp., *Oscillibacter* spp., *Escherichia* spp., *Clostridium_sensu_stricto* spp., *Streptococcus* spp., *Sphingomonas* spp., *Terrisporobacter* spp., *Fusobacterium* spp., *Pseudomonas* spp., and *Bacteroides* spp.. At the time of the first diarrhea after birth, the dominant genera shifted to *Fusobacterium* spp., *Clostridium_XlVa* spp., *Bacteroides* spp., *Akkermansia* spp., *Subdoligranulum* spp., *Roseburia* spp., *Alistipes* spp., *Lactobacillus* spp., *Odoribacter* spp., and Blautia spp. On day 30, the major microbiota shifted to *Akkermansia* spp., *Oscillibacter* spp., *Clostridium_XlVa* spp., *Bacteroides* spp., *Odoribacter* spp., *Paraprevotella* spp., *Eubacterium* spp., *Phascolarctobacterium* spp., *Prevotella* spp., *Ruminococcus* spp., and *Campylobacter* spp. On day 60, the main microbiota were *Clostridium_XlVa* spp., *Akkermansia* spp., *Bacteroides* spp., *Oscillibacter* spp., *Eubacterium* spp., *Fusobacterium* spp., *Fibrobacter* spp., *Treponema* spp., and *Prevotella* spp. On day 90, the primary microbiota comprised *Fibrobacter* spp., *Clostridium_XlVa* spp., *Treponema* spp., *Olivibacter* spp., *Prevotella* spp., *Akkermansia* spp., *Oscillibacter* spp., and *Ruminococcus* spp. On day 120, the main microbiota were *Clostridium_XlVa* spp., *Fibrobacter* spp., *Olivibacter* spp., *Treponema* spp., *Prevotella* spp., *Oscillibacter* spp., and *Ruminococcus* spp.

As shown in Figure 8, the top twenty genera of the intestinal microbiota in foals showed varying changes from day 0 to day 120 after birth. On day 0, compared with the NC group, *Phascolarctobacterium* spp. in the GY group was significantly reduced (*p* < 0.05; Figures 8A1, A2). During the first diarrhea episode, *Odoribacter* spp. and *Akkermansia* spp. in the MY group were significantly higher than in the NC and GY groups (*p* < 0.05; Figures 8B1, B2, C1, C2). *Streptococcus* spp. in the GY group was higher than that in the MY group (Figures 8D1, D2). On day 30, compared with the NC group, *Ruminococcus* spp. in the MY group was significantly increased (*p* < 0.05; Figures 8E1, E2). On day 90, *Eubacterium* in the GY group was significantly higher than that in the MY group (*p* < 0.05; Figures 8F1, F2).

### 3.13 Functional analysis of gut microbiota

Functional prediction based on the KEGG database revealed that the average relative abundance of the major functional categories in the intestinal microbiota of foals from day 0 to day 120 was dominated by Metabolism (80.04%), followed by Genetic Information Processing (14.89%), Cellular Processes (4.91%), Environmental Information Processing (2.62%), Organismal Systems (0.46%), and Human Diseases (0.56%).

A total of 35 secondary pathways were predicted, and the top ten intestinal microbiota functions in foals from day 0 to day 120 were Carbohydrate Metabolism (13.57%), Amino Acid Metabolism (12.59%), Metabolism of Cofactors and Vitamins (12.42%), Metabolism of Terpenoids and Polyketides (10.24%), Metabolism of Other Amino Acids (7.04%), Replication and Repair (6.17%), Lipid Metabolism (5.55%), Energy Metabolism (5.50%), and Glycan Biosynthesis and Metabolism (3.91%).

**Figure 9:**
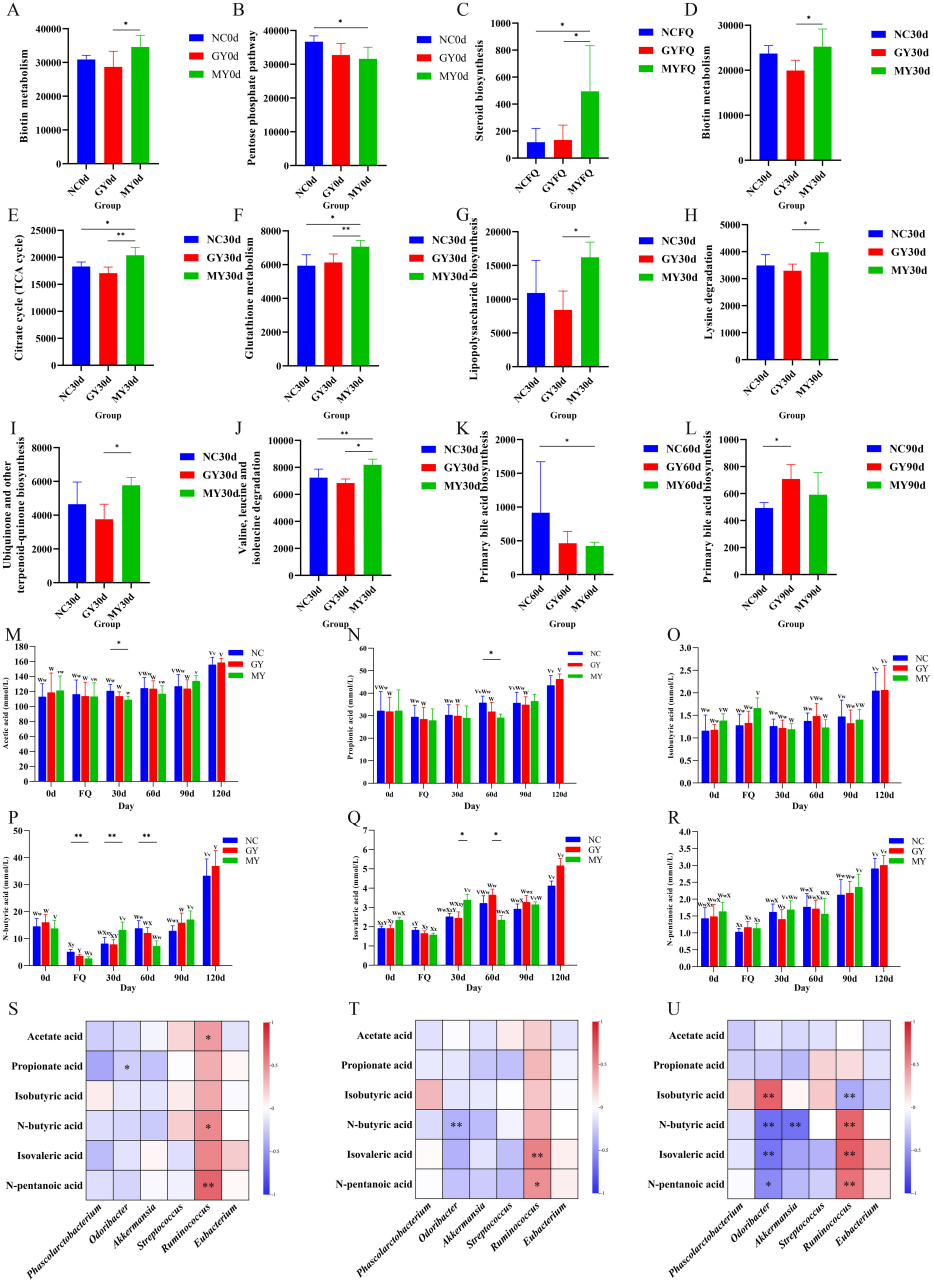
Differential analysis of predicted intestinal microbiota functions in foals from day 0 to day 120 **(A–L)**: **(A, B)** Day 0 (birth); **(C)** first occurrence of diarrhea; **(D–J)** Day 30; **(K)** Day 60; **(L)** Day 90. Effect of TSPSS supplementation on short-chain fatty acid (SCFAs) in foals **(M–R)**: **(M)** Acetic acid (mmol/L); **(N)** Propionic acid (mmol/L); **(O)** Isobutyric acid (mmol/L); **(P)** N-butyric acid (mmol/L); **(Q)** Isovaleric acid (mmol/L); **(R)** N-pentanoic acid (mmol/L). Heatmap showing correlation between SCFAs and gut bacterial genera **(S–U)**: **(S)** NC, **(T)** GY, and **(U)** MY groups. Significant differences within groups (*p < 0.05, **p < 0.01). Significant differences between groups (^v − z^*p* < 0.05, ^V − Z^*p* < 0.01).

At the tertiary pathway level, several significant differences in predicted functional abundances were observed across groups and time points (Figures 9A–L). On day 0 (birth), compared to the NC group, the Pentose Phosphate Pathway in the MY group was significantly reduced (*p* < 0.05), while compared to the GY group, Biotin Metabolism in the MY group was significantly increased (*p* < 0.05). During the first diarrhea episode, Steroid Biosynthesis in the MY group was significantly higher than that in the NC and GY groups. On day 30, compared to the NC group, Citrate Cycle, Glutathione Metabolism, and Valine, Leucine, and Isoleucine degradation in the MY group were significantly increased (*p* < 0.05). Compared to the GY group, Biotin Metabolism, Lipopolysaccharide Biosynthesis, Lysine Degradation, and Ubiquinone and other Terpenoid-Quinone Biosynthesis in the MY group were significantly elevated (*p* < 0.05), with the Citrate Cycle, Glutathione Metabolism, and Valine, Leucine, and Isoleucine degradation showing extremely significant increases (*p* < 0.01). On day 60, compared to the NC group, Primary Bile Acid Biosynthesis in the MY group was significantly reduced (*p* < 0.05). On day 90, compared to the NC group, Primary Bile Acid Biosynthesis in the GY group was significantly increased (*p* < 0.05). These predictions should be regarded as a basis for generating hypotheses and should be validated in future studies through targeted metabolomic or transcriptomic analyses.

### 3.14 Effect of TSPSS supplementation on SCFA production in foals

A significant effect was observed on fecal SCFAs concentrations in the foals under the main effects of developmental stage and grouping, as well as under their interaction (Supplementary Table S6). Feces samples were collected and analyzed to investigate the effect of TSPSS supplementation on the formation of SCFAs in foals. Compared with the NC group, during the first diarrhea, the levels of N-butyric acid in the MY groups were significantly lower (*p* < 0.01; Figure 9). On day 30, the concentration of acetic acid in the feces of MY group foals was significantly lower (*p* < 0.05), while N-butyric acid was significantly higher than that in NC group (*p* < 0.01). Compared with the MY group, on day 30, the levels of N-butyric acid and isovaleric acid in the feces of GY group were significantly lower (*p* < 0.05), while the acetic acid level in the MY group was significantly lower and N-butyric acid was significantly higher (*p* < 0.05 and *p* < 0.01, respectively). On day 60, the levels of propionic acid and N-butyric acid in the feces of MY group were significantly reduced (*p* < 0.05 and *p* < 0.01, respectively), while isovaleric acid in the GY group was significantly higher (*p* < 0.05). SCFAs, primarily produced through gut microbiota fermentation, are known to regulate gastrointestinal motility and help maintain intestinal homeostasis and barrier function (Figures 9M–R).

### 3.15 Correlation between SCFAs and intestinal bacteria at the genus level in foals

As shown in Figures 9S–U, Spearman correlation analysis revealed significant associations between fecal SCFA concentrations and the relative abundances of selected genera across the three groups. In the NC group, propionic acid and *Phascolarctobacterium* spp. showed a significant negative correlation with (*p* < 0.05). Acetic acid and N-butyric acid were significantly positively correlated with *Ruminococcus* spp. (*p* < 0.05). Moreover, N-pentanoic acid exhibited a highly significant positive correlation with *Ruminococcus* spp. (*p* < 0.01). These results suggest that acetic acid, N-butyric acid, and N-pentanoic acid may promote the relative abundance of *Ruminococcus* spp. in the NC group.

In the GY group, N-butyric acid was highly negatively correlated with *Odoribacter* spp. (*p* < 0.01), while isovaleric acid and N-pentanoic acid were significantly positively correlated with *Ruminococcus* spp. (*p* < 0.01 and *p* < 0.05, respectively). These findings suggest that TSPSS supplementation in the GY group may promote the relative abundance of *Ruminococcus* spp., while inhibiting the relative abundance of *Odoribacter* spp.

In the MY group, isobutyric acid exhibited a highly significant positive correlation with *Odoribacter* spp. (*p* < 0.01), while N-butyric acid, isovaleric acid, and N-pentanoic acid were significantly negatively correlated (*p* < 0.05). Additionally, N-butyric acid showed a highly significant negative correlation with *Akkermansia* spp. (*p* < 0.01). Isobutyric acid was highly negatively correlated with *Ruminococcus* spp., while N-butyric acid, isovaleric acid, and N-pentanoic acid were highly positively correlated with *Ruminococcus* spp. (*p* < 0.01).

## 4 Discussion

Body size and weight are crucial indicators for assessing the growth and development of foals. During the suckling period, foals exhibit significant growth acceleration, particularly in body weight, accompanied by substantial skeletal development These changes are closely associated with increases in body weight, with prominent alterations in body dimensions being especially evident during this phase (Aranda, 2021). SOD, MDA, and T-AOC are commonly used indicators reflecting the antioxidant capacity of the body, which is essential for maintaining the stability and health of foal growth and development (Zhang L. Y. et al., 2021). IL-6, IFN-γ, and TNF-α play crucial roles in resisting external environmental insults, responding to infections, and regulating cellular immunity (Li et al., 2022; Zhang X. et al., 2021). However, research has shown that the overexpression of IL-6 and IFN-γ can lead to immune diseases and even promote tumorigenesis, affecting growth and development (Tanaka et al., 2014). The activation of TNF-α can trigger inflammatory responses and lead to critical conditions such as shock. In contrast, IL-10, an anti-inflammatory cytokine, primarily participates in regulating inflammatory and cellular immune responses (Zhao et al., 2020). Therefore, controlling the levels of these inflammatory cytokines is essential for ensuring the health of foals.

Immunoglobulins are important indicators for assessing animal immune function in animals. Previous studies have shown that supplementation of lactating mares with bamboo leaf extracts significantly enhances the immune performance of their foals, suggesting that herbal feed additives may strengthen the immune system and exert positive effects on foal growth and development (Zhang et al., 2022). GH plays a central role in regulating individual growth through binding to its receptors. Activation of GH receptors stimulates the structural production of IGF-I, a peptide similar to insulin, which is primarily synthesized by the liver but is also secreted by other organs (Li et al., 2021). In addition to its classical regulatory functions, insulin also exhibits significant growth-promoting effects. Thyroid hormones primarily function by promoting metabolism, increasing oxygen consumption, and facilitating tissue differentiation, growth, and maturation (Miller et al., 2022). Thyroid hormones are particularly important during infancy, especially within the first 4 months after birth, and play a primary role in promoting the growth and development of bones, the brain, and reproductive organs. Additionally, thyroid hormones synergize with growth hormones to regulate growth rates (Eng and Lam, 2020). T3 and T4 are the primary hormones secreted by the thyroid gland, with T3 exhibiting five times greater activity than T4. T4 is converted into the more active T3 in the liver and other tissues, a process regulated by multiple factors. Only free T3 possesses metabolic activity and can accurately reflect thyroid function and its impact on physiological functions. Therefore, T3 and T4 influence various organs by affecting cell metabolism and promoting protein synthesis, comprehensively regulating the body's metabolic activities and having a significant impact on the development of the skeletal and nervous systems (Zamwar and Muneshwar, 2023).

In this study, we measured E_2_ and P_4_ levels in mares at different stages of pregnancy and found that during early gestation, mares have low E_2_ levels, which gradually increase as pregnancy progresses (Liebmann et al., 2022). Previous studies have shown that E_2_ interacts with other hormones and signaling molecules to maintain the health of mares and fetal development (Satué et al., 2021). De Amorim et al. (2023) reported that early and mid-gestation are critical periods for embryonic development, during which the placenta begins to enhance its hormone synthesis and secretion capabilities. P_4_ is another crucial hormone for maintaining pregnancy, promoting endometrial thickening and placenta formation (Muro et al., 2022). Pappa et al. (2022) discovered that P_4_ supported fetal development during mid-gestation, but was unaffected by supplementary feeding. According to previous research (Porto et al., 2023), P_4_ levels decreased significantly in late gestation as the fetus was prepared for birth, with an increase in hormones such as oxytocin suppressing P_4_ synthesis. Therefore, P_4_ levels should decrease in mares during late gestation, as elevated P_4_ levels may increase the risk of immune system defects and diseases in newborn foals (Wang et al., 2023). The results of body measurements and weight changes in foals showed that the growth patterns of foals differ in both the short and long term, with the early stages of foal growth being particularly crucial for their lifelong development (Zhang C. et al., 2023). The experimental results of Wu et al. (2022) indicated that supplementing the diet of pregnant sows with L-arginine significantly increases the birth weight of piglets. Similar studies have also shown that feeding pregnant sows TCM herbs has the same effect on improving birth weight (Carroll et al., 2002). These studies collectively demonstrate that specific nutritional or pharmacological interventions during pregnancy can have a positive impact on fetal growth and development. Our findings reveal that adjusting the supplementation frequency in pregnant mares influences the growth performance of their foals. Specifically, intermittent supplementation every other month significantly increased the weight and chest circumference of foals shortly after birth, while continuous supplementation appeared more suitable for long-term growth.

Our findings also demonstrate that dynamic alterations in serum concentrations of key inflammatory cytokines, IL-6, IFN-γ, and TNF-α, and the anti-inflammatory IL-10, serve as biomarkers of systemic immune modulation and homeostatic responses (Lima Chechin Catussi et al., 2022; Cognie et al., 2023). Studies have shown that the addition of flavonoid compounds to feed can reduce inflammation by lowering TNF-α levels (Kodbe et al., 2022). Du et al. (2016) reported that the levels of IFN-γ and TNF-α in the body significantly increased as growth and development progressed, and this trend intensified over time. Consistent with these observations, this study demonstrates that TSPSS supplementation in pregnant mares shows a regulatory effect on immune markers in their foals. And this effect becomes more pronounced as the foals grow. However, during the first 90 days of life, the observed patterns may reflect the physiological need for balanced anti-inflammatory responses and tissue repair during the early growth and development of foals after birth (Akyol et al., 2024).

The results of this study suggest that intermittent TSPSS supplementation every other month (bimonthly) is more effective than monthly supplementation in modulating serum antioxidant marker levels and immune parameters in foals. SOD, a key antioxidant enzyme, plays a critical role in maintaining intracellular homeostasis and protecting cells from oxidative stress-induced damage (Hu et al., 2023). MDA, which serves as an oxidative marker in the body, is negatively correlated with SOD (Kumar et al., 2017). A study conducted by Duan et al. (2017) revealed that elevated T-AOC capacity indicated increased antioxidant enzyme activity within the body, enabling improved clearance of harmful oxidants, such as free radicals, and thereby protecting cells from oxidative stress damage. Published research suggests that as young animals grow and their environment changes, the production of free radicals and oxidative damage within the body increases, posing a threat to their healthy development (Hu et al., 2023). However, as shown in our results, both intermittent and continuous supplementation with TSPSS can enhance systemic antioxidant capacity and mitigate free radical-mediated oxidative damage. Based on the observed changes in serum antioxidant marker levels and serum immune parameters, it can be hypothesized that the active ingredients in TSPSS enhance T-AOC by increasing the level of antioxidants such as SOD and peroxidase in foals (Enstad et al., 2021; Peugnet et al., 2016). Therefore, the potential regulatory role of TSPSS may be related to intermittent supplementation. It may help foals establish and strengthen their immune systems, support overall health, and promote optimal growth and development.

Previous studies have shown that the content of acquired immunoglobulins in the bloodstream of foals shortly after birth reflected the immune strength and disease resistance of the body (Nieman and Chan, 2022). It has been reported that feeding TSPSS to pregnant dams can effectively increase the levels of IgA, IgG, and IgM in their offspring and enhance immune function (Liu, 2020). The colostrum from mares fed TSPSS is rich in immunoglobulins and can quickly help the newborn foals establish their immune defense system (Viola et al., 2022). At birth, gut closure occurs so that foals are able to absorb intact Ig's by passive transfer, but normal metabolism begins about 24 h after birth and the Ig's are digested as any other large protein. Therefore, foals need to ingest sufficient amounts of colostrum quickly to enhance their immune defense system and protect themselves from external pathogens (Aoki et al., 2020). Given that there is a half-life for Ig metabolism in animals (Harmsen et al., 2024), the Ig levels in foals aged 30–90 days tend to decrease significantly. However, in this study, Ig content in the GY group increased again at 60 days, suggesting that foals in this group were able to effectively increase their Ig levels to adapt to external environment challenges. In the early stages of growth and development, the immune system of foals is not fully established, resulting in relatively low concentrations of IgA and IgM (Price et al., 2017). Nevertheless, studies have shown that nutritional and pharmacological supplementation during pregnancy can effectively increase the immune status of foals (Coverdale et al., 2015; Fehrenkamp et al., 2019). It has also been found that the Ig content in colostrum is influenced by the season of parturition. Shorter daylight hours and reduced lactation in autumn and winter lead to less dilution of milk and higher Ig content, whereas the opposite is true in spring and summer (Costa et al., 2021). Since the foals in GY group in this study were born from March to April, while those in the NC and MY groups were born from April to June. This seasonal difference in parturition timing may partially explain the relatively lower Ig levels observed in the MY group.

Collectively, these findings demonstrate that dynamic fluctuations in serum growth factor profiles during foal development may underpin growth-related physiological adaptations, with intermittent TSPSS supplementation exhibiting superior efficacy in modulating these pathways to enhance somatic and developmental outcomes. GH and IGF-I levels are often closely related to growth and development, metabolic status, and aging in animals (Yakar and Isaksson, 2016). In animals, IGF-I levels are often closely related to growth and development, metabolic status, and aging (Tang et al., 2022). The thyroid hormones, T_3_ and T_4_, have significant impacts on nutritional metabolism, thermoregulation, the nervous system, and the immune system (Steinhoff et al., 2019). Increasing the insulin concentration can increase the number of GH receptors in the body, thereby promoting the synthesis and secretion of GH (Glynn et al., 2021). The findings of this study suggest that foals aged 0–90 days are in a peak growth period, further corroborating the long-term effectiveness of supplementary feeding with TSPSS in enhancing the growth rate of foals and providing optimal support for their growth and development.

Correlation analysis revealed associations between serum hormone levels and growth performance indicators in foals. These results suggest that as foals grow and develop, their bodies gradually mature, enhancing their adaptability to the external environment and ultimately leading to a gradual decrease in the demand for immune factors. Overall, the results indicate that the regulatory role of TSPSS may be associated with intermittent supplementation and may contribute to improved foal growth performance. When mares are supplemented with TSPSS, active ingredients such as ginsenosides, flavonoids, and polysaccharides can enhance the growth and development of foals while elevating IL-6 levels. SOD protects cells from oxidative damage, further promoting the growth and development of foals (Liu L. et al., 2022). The significant negative correlations observed between T-AOC, IgA, IgG, and IgM with body weight, body height, body oblique length, chest circumference, and cannon circumference in all three groups of foals can be attributed to the gradual maturation of the foals. This maturation enhances their adaptability to the external environment, ultimately leading to a gradual decrease in the demand for immune factors, thus explaining the negative correlation (Shi et al., 2020).

Gut microbiota analysis in this study revealed significant alterations in microbial composition and diversity across the experimental groups and time points. Previous studies have indicated (Wang et al., 2021) that the intestinal microbiota of newborn animals is in the initial stage of colonization, resulting in a higher number of OTUs. As the intestinal microbiota gradually stabilizes, the number of OTUs decreases. Diarrhea may lead to an imbalance in the intestinal microbiota, reducing the number of microorganisms, and thereby lowering the number of OTUs. In studies on animals such as pigs, horses, and cattle, Firmicutes and Bacteroidetes have been identified as the dominant microbiota in their GI tracts (Ley et al., 2008). Firmicutes, the primary gut bacterial group, can metabolize fiber and extract more energy from fiber-rich diets to meet the growth demands of young animals (Hamaker and Tuncil, 2014). Additionally, the strength of digestive ability is closely related to the composition and abundance of intestinal microbes (Jiao et al., 2014). The other major phylum, Bacteroidetes (aka Bacteroidota), is also indispensable for herbivores to digest the complex carbohydrates in roughage (Spence et al., 2006).

On day 30 after birth, foals from the GY group exhibited a significantly lower abundance of Chlamydiae and a significantly higher relative abundance of Synergistetes compared with the NC group. These findings suggest that maternal TSPSS supplementation during pregnancy may contribute to changes in the composition and relative abundance of the foal gut microbiota at the phylum level during growth. As foals age, the structure and composition of their intestinal microbiota change, primarily influenced by changes in dietary patterns, increased food intake, and changes in their living environment. This leads to an upward trend in the stability, diversity, and complexity of the intestinal microbiota. This change helps establish a relatively stable and beneficial internal environment in the digestive tract of foals, supports their healthy growth (Gomez et al., 2023). Different supplementation frequencies of TSPSS in pregnant mares also have a significant impact on the intestinal microbiota structure of foals. On day 0(birth), the abundance of *Phascolarctobacterium* spp. in the GY group was significantly lower than that in the NC group. However, by day 30 after birth, the abundance of Ruminococcus in the MY group was significantly higher than that in the NC group. *Phascolarctobacterium* spp., an obligate anaerobic bacterium commonly found in the gut, plays a key role in maintaining intestinal homeostasis by converting succinate to propionate, a short-chain fatty acid that supports colonic epithelial health and energy supply. *Ruminococcus* spp., a major genus within the Firmicutes phylum, is well-known for its involvement in fiber degradation, cellulose breakdown, and methane production.

Gestational feeding may indirectly affect the intestinal microbiota structure of foals at birth by influencing the intestinal microbiota community of mares (Ma et al., 2019). During the first episode of diarrhea, foals in the MY group exhibited significantly higher relative abundances of *Odoribacter* spp. and *Akkermansia* spp. compared with both NC and GY groups. *Odoribacter* spp. and *Akkermansia* spp. are considered to be related to intestinal health and diarrhea occurrence in some studies. Related research suggests that *Akkermansia* spp. may have potential benefits in preventing and treating metabolic diseases such as obesity, diabetes, and inflammation (Macchione et al., 2019). It helps maintain intestinal health, reduces inflammatory responses, and may affect host energy metabolism by regulating intestinal mucosal barrier function, influencing the intestinal environment, and immune responses (Zhu et al., 2021). Studies have found that a decrease in the number of *Odoribacter* spp. is associated with the onset of metabolic diseases, especially colitis and non-alcoholic fatty liver disease. However, despite the high abundance of *Odoribacter* spp., it does not appear to affect colon health, likely due to its relatively low overall number in the intestinal microbiota (Yi et al., 2023). Additionally, the abundance of *Streptococcus* spp. in the GY group was significantly higher than that in the MY group. On day 90 after birth, the abundance of *Eubacterium* spp. in the GY group was significantly higher than that in the MY group. *Eubacterium* spp. is a microorganism essential for animal health and intestinal function, primarily involved in various physiological processes, including nutrient digestion and metabolism, immune system regulation, and intestinal barrier function protection (Han et al., 2023).

Functional prediction analysis of the gut microbiota revealed significant shifts in predicted metabolic pathways and functional profiles across groups and time points, which may influence host physiological states or disease susceptibility in foals. Alharthi et al. (2019) reported that increasing intestinal supply to late-gestation dairy cows resulted in the synthesis of phosphatidylcholine and the promotion of energy metabolism in calves, indicating their importance for the growth and development of young animals. On day 0 (birth), the predicted activity of the pentose phosphate pathway in the MY group was significantly lower than that in the NC group, whereas biotin metabolism was significantly higher than in the GY group. During the first episode of diarrhea, steroid biosynthesis in the MY group was significantly higher than in the other two groups. By day 30, the MY group showed a significant increase in several key pathways, including the citrate cycle, glutathione metabolism, and valine, leucine, and isoleucine degradation, compared with the NC group. Compared with the GY group, the MY group also showed significant increases in aminoacyl biosynthesis, arginine and proline metabolism, and cysteine and methionine metabolism. These functional enhancements are likely related to the continuous TSPSS treatment of mares in the MY group. On days 60 and 90, the MY group showed a significant decrease in the predicted activity of primary bile acid biosynthesis, while the GY group showed a significant increase. Primary bile acids are metabolic products of cholesterol breakdown in the liver and participate in lipid digestion and absorption. Supplementing TSPSS during gestation may affect the digestive system and lipid metabolism of foals (Liu X. et al., 2022). It is important to emphasize that these findings are computational inferences based on 16S rRNA data rather than direct measurements of metabolic functions. Future studies using metagenomics, metatranscriptomics, or targeted metabolomics are needed to validate these predicted functional changes.

SCFAs are critical mediators of gut microbiota-host interactions, and significant changes in SCFA profiles were observed in foals from mares supplemented with TSPSS. Relevant research indicates that SCFAs regulate gastrointestinal motility, primarily produced by probiotics, and maintain intestinal function stability. A decrease in acetic acid may create an environment more suitable for the growth of certain pathogens (Chen et al., 2024). However, the increase in N-butyric acid may be a compensatory response to the decrease in acetic acid, aiming to maintain intestinal environmental stability. Changes in the fecal levels of acetic, propionic and butyric acids in the foals from the MY group suggest that TSPSS supplementation may influence gut microbial metabolism. Furthermore, monthly supplementation may also alter the composition and activity of the foal gut microbiota, which may influence the production and metabolism of short-chain fatty acids. The significant decrease in propionic acid and N-butyric acid in the MY group on day 60 suggests that higher frequencies of TSPSS supplementation have a prolonged impact on the production of SCFAs in foals.

The correlation analysis between fecal SCFA concentrations and differentially abundant genera at genus level revealed several significant associations. Studies have found that isovaleric acid exhibited antibacterial, anti-inflammatory, and antioxidant activities, which are crucial for protecting animal intestinal health (Xue et al., 2020). In this study, the fecal isovaleric acid level in the GY group was significantly higher than that in the MY group at different time points, indicating a higher abundance of bacteria capable of synthesizing isovaleric acid in the intestinal tract of the GY group. *Ruminococcus* spp., one of the SCFA-producing bacteria, plays an important role in intestinal health and function. A significant positive correlation was observed between *Ruminococcus* spp. and SCFAs in the NC and GY groups, suggesting a synchronous growth trend in the abundance of *Ruminococcus* spp. and SCFA production in the intestinal tract during the growth and development of foals. This positive correlation suggests that *Ruminococcus* spp. may be involved in SCFA production in foals. These findings also suggest that TSPSS supplementation may help maintain a stable intestinal environment and support nutrient absorption and utilization, both of which may benefit foal growth and development. However, previous studies have shown that SCFA production often decreases in various disease states, accompanied by an increase in potentially pathogenic or opportunistic genera, such as *Odoribacter* spp. (Liao et al., 2023). Consistent with this finding, the results in this study showed a negative correlation between SCFAs and *Odoribacter* abundance in the NC and GY groups. In the MY group, however, isobutyric acid exhibited a highly significant positive correlation with *Odoribacter* spp., while N-butyric acid, isovaleric acid, and N-pentanoic acid showed highly significant negative correlations. These findings indicate that continuous TSPSS supplementation affected this trend in foals.

*Akkermansia* spp. releases SCFAs by degrading mucins, which regulate the immune and inflammatory responses of the host, thus protecting intestinal barrier integrity and overall gut health. SCFAs inhibit histone deacetylases, regulate the NF-κB pathway, reduce the production of inflammatory factors, nourish intestinal epithelial cells, promote their proliferation and differentiation, enhance mucosal barrier function and stabilize intestinal microenvironment (Liao et al., 2023). The results of this study showed a nearly negative correlation between SCFAs and *Akkermansia* spp. in the intestinal tracts of foals in all three groups. Among them, propionic acid and N-butyric acid in the MY group showed a significant negative correlation, possibly due to the continuous adaptation of foals to the external environment during their growth and development, resulting in temporary unstable intestinal microbiota formation and SCFA production.

We acknowledge that this study has two main limitations. First, the relatively small sample size (*n* = 4 per group) may reduce the statistical power, particularly for gut microbiota analysis, which is subject to high individual variation. This was mainly due to the strict selection criteria for purebred English Thoroughbred pregnant mares. Second, although husbandry conditions were standardized, seasonal differences in foaling time among groups may have slightly influenced maternal colostrum production and early gut microbial colonization in foals. In addition, the repeated-measures nature of the data suggests that mixed-effects models should be further applied and refined in future studies. Identifying the active components of TSPSS and establishing dose–response relationships will also be important priorities for future research. Further pharmacodynamic studies will be needed to investigate the mechanisms of action of TSPSS. Future studies should therefore include larger sample sizes, tighter control of foaling time and seasonal factors, and a more rigorous design to minimize these potential confounding effects. It should also be noted that all findings in this study are based on correlations observed in the data. The causal relationship between TSPSS supplementation and changes in foal phenotype, as well as the possible mediating role of the gut microbiota-metabolism axis, requires further validation.

## 5 Conclusions

This study showed that maternal TSPSS supplementation was associated with changes in serum E2 and P4 levels in pregnant mares, as well as differential expression of genes related to placental development and nutrient transport. These changes may be related to the improved growth of foals after birth. Gestational TSPSS supplementation was also associated with increased body weight, body height, body length, and chest circumference in foals, alongside higher levels of IL-6, IFN-γ, TNF-α, GH, T3, T4, IGF-I, insulin, IgA, IgG, SOD, and T-AOC, and lower levels of MDA. As the foals grew and developed, positive correlations were observed between body weight and IL-6 and IFN-γ levels and between body weight and height, whereas negative correlations were found between body weight and SOD, T-AOC, immunoglobulins, T_3_, and T_4_. Bi-monthly TSPSS supplementation in mares improved the intestinal microbiota composition in foals, characterized by increased relative abundance of *Synergistetes* spp., decreased abundance of *Chlamydiae* spp. and *Phascolarctobacterium* spp., and reduced N-butyric acid levels during episodes of diarrhea. Monthly supplementation, in comparison, increased abundance of *Odoribacter* spp. and *Akkermansia* spp., and enhanced predicted metabolic functions such as amino acids synthesis and related pathways. Concurrently, changes in the intestinal microbiota of foals also affected the production of SCFAs. In lactating mares, TSPSS supplementation increased the abundance of Firmicutes and Treponema, reduced the abundance of *Succinivibrionaceae* spp., and altered the intestinal flora composition during lactation. Collectively, these results demonstrate that dietary supplementation of TSPSS in pregnant mares exerts positive effects on the growth and development of suckling foals. Overall, the effects of bi-monthly dietary supplementation of TSPSS were more beneficial than monthly supplementation and may effectively reduce the cost of practical application.

## Data Availability

The datasets presented in this study can be found in online repositories. The names of the repository/repositories and accession number(s) can be found in the article/Supplementary material.
