# Supplementary material for: Maternal Taishan Panshi San supplementation is associated with improved foal growth performance, accompanied by alterations in gut microbiota and metabolic profiles
Source: Front Microbiol. 2026 Apr 20;17:1787938. doi: 10.3389/fmicb.2026.1787938 (PMC13136102; doi:10.3389/fmicb.2026.1787938)
Supplement: Supplementary file 1 [file Table_1.docx]

**Supplementary Materials**

**Table S1.** Identified compounds in TSPSS under positive ion mode.

| RT  (min) | Ingredient name | Formula | Adduct type | Observed  (m/z) | Error  (ppm) | Compounds | CAS No. |
| --- | --- | --- | --- | --- | --- | --- | --- |
| 4.25 | Verbascoside | C_29_H_36_O_15_ | M+NH_4_, M+Na, M+H | 642.2385 | -1.24 | Phenolic acids and derivatives | 61276-17-3 |
| 7.69 | Hoduloside IV | C_47_H_76_O_18_ | M+NH_4_, M+Na, M+H | 946.5355 | -1.57 | Terpenoids | 146445-91-2 |
| 7.26 | Xenognosin B | C_16_H_12_O_5_ | M+CH_3_OH+H | 317.1017 | -0.84 | Flavonoids | 1890-99-9 |
| 9.47 | Limonin | C_26_H_30_O_8_ | M+H, M+NH_4_, M+Na, M+K | 471.2012 | -0.40 | Terpenoids | 1180-71-8 |
| 4.49 | Narirutin | C_27_H_32_O_14_ | M+H, M+Na, M+H-H_2_O, M+H-2H_2_O | 581.186 | -0.91 | Flavonoids | 14259-46-2 |
| 4.74 | Neohesperidin | C_28_H_34_O_15_ | M+H, M+NH_4_, M+Na, M+H-H_2_O, M+H-2H_2_O | 611.196 | -1.70 | Flavonoids | 13241-33-3 |
| 1.11 | L-Leucine | C_6_H_13_NO_2_ | M+H | 132.1021 | 1.65 | Amino acids and derivatives | 61-90-5 |
| 10.21 | Tangeretin | C_20_H_20_O_7_ | M+H, M+Na | 373.1279 | -0.84 | Flavonoids | 481-53-8 |
| 11.96 | Nobiletin | C_21_H_22_O_8_ | M+H, 2M+Na, M+Na, M+K | 403.1384 | -0.89 | Flavonoids | 10236-47-2;478-01-3 |
| 13.50 | Linoleic acid | C_18_H_32_O_2_ | M+H-H_2_O, M+NH_4_, M+H-2H_2_O, M+H | 298.2738 | -0.85 | Lipids | 506-21-8;60-33-3 |
| 0.58 | Canavanine | C_5_H_12_N_4_O_3_ | M+H, M+Na, M+K, M+H-H_2_O | 177.0982 | -0.13 | Amino acids and derivatives | 543-38-4 |
| 13.48 | Dibutyl Phthalate | C_16_H_22_O_4_ | M+H, M+Na | 279.1589 | -0.65 | Phenolic acids and derivatives | 84-74-2 |
| 0.44 | Linalool | C_10_H_18_O | M+H-H_2_O | 137.1326 | 0.92 | Terpenoids | 78-70-6 |
| 3.52 | Loganin | C_17_H_26_O_10_ | M+H-H_2_O, M+H, M+NH_4_, M+Na, M+K | 408.186 | -1.03 | Terpenoids | 18524-94-2 |
| 6.71 | Ursonic acid | C_30_H_46_O_3_ | M+H-H_2_O, M+H | 455.3514 | -1.18 | Terpenoids | 6246-46-4 |
| 5.31 | Betulin | C_30_H_50_O_2_ | M+2Na-H | 487.3486 | -8.34 | Terpenoids | 473-98-3 |
| 11.46 | Heptadecanoic acid | C_17_H_34_O_2_ | M+NH4 | 288.2894 | -1.26 | Lipids | 506-12-7;3546-17-6;106182-29-0 |
| 3.23 | Zizybeoside I | C_19_H_28_O_11_ | M+NH4, M+Na | 450.1967 | -0.71 | Carbohydrates and derivatives | 76819-28-8 |
| 7.69 | Maslinic acid | C_30_H_48_O_4_ | M+H-2H_2_O, M+H, M+H-H_2_O | 437.3409 | -1.17 | Terpenoids | 4373-41-5 |
| 4.78 | Berberine | C_20_H_18_NO_4_^+^ | M+ | 336.1224 | -3.51 | Alkaloids and derivatives | 2086-83-1 |
| 1.06 | Guanosine | C_10_H_13_N_5_O_5_ | M+H, M+Na, 2M+Na, 2M+H | 284.099 | 0.12 | Nucleotides and derivatives | 118-00-3 |
| 8.71 | Ligustilide | C_12_H_14_O_2_ | M+H | 191.1065 | -0.59 | Others | 4431-01-0 |
| 10.29 | Curdione | C_15_H_24_O_2_ | M+H-H_2_O, 2M+H, M+Na, M+H-2H_2_O | 473.3623 | -0.44 | Terpenoids | 13657-68-6 |
| 10.49 | Palmitic acid | C_16_H_32_O_2_ | M+NH4 | 274.2737 | -1.30 | Lipids | 21096 |
| 0.72 | Nicotinic acid | C_6_H_5_NO_2_ | M+H | 124.0396 | 2.52 | Others | 583-08-4;59-67-6 |
| 3.09 | Ferulic acid | C_10_H_10_O_4_ | M+H-H_2_O | 177.0547 | 0.52 | Phenolic acids and derivatives | 537-98-4 |
| 7.86 | Myristic acid | C_14_H_28_O_2_ | M+NH4 | 246.2426 | -0.65 | Lipids | 544-63-8 |
| 6.03 | Tectorigenin | C_16_H_12_O_6_ | M+H | 301.0703 | -1.31 | Flavonoids | 548-77-6 |
| 2.87 | Apigenin | C_15_H_10_O_5_ | M+CH_3_OH+H | 303.0862 | -0.38 | Flavonoids | 520-36-5 |
| 9.78 | Glycyrrhetinic acid | C_30_H_46_O_4_ | M+H-H_2_O, M+H | 453.3359 | -0.84 | Terpenoids | 471-53-4;1449-05-4;471-53-4 |
| 7.17 | Hispidulin | C_16_H_12_O_6_ | M+H | 301.0704 | -1.02 | Flavonoids | 1447-88-7 |
| 4.14 | Naringenin | C_15_H_12_O_5_ | M+H | 273.0754 | -1.24 | Flavonoids | 480-41-1 |
| 11.08 | Semilicoisoflavone B | C_20_H_16_O_6_ | M+H, M+Na | 353.1016 | -1.00 | Flavonoids | 129280-33-7 |
| 3.72 | Schaftoside | C_26_H_28_O_14_ | M+H | 565.1548 | -0.60 | Flavonoids | 207461-10-7;51938-32-0 |
| 7.51 | Licoflavonol | C_20_H_18_O_6_ | M+H | 355.1172 | -1.29 | Flavonoids | 60197-60-6 |
| 12.33 | Gancaonin B | C_21_H_20_O_6_ | M+H-H_2_O | 351.1211 | -4.34 | Flavonoids | 124596-86-7 |
| 8.21 | Isoliquiritigenin | C_15_H_12_O_4_ | M+H | 257.0806 | -1.00 | Flavonoids | 961-29-5 |
| 11.13 | Glycycoumarin | C_21_H_20_O_6_ | M+H, M+Na, M+CH_3_OH+H | 369.133 | -0.74 | Flavonoids | 94805-82-0 |
| 5.01 | Liquiritin | C_21_H_22_O_9_ | M+H | 419.1333 | -0.81 | Flavonoids | 551-15-5 |
| 4.00 | Violanthin | C_27_H_30_O_14_ | M+H | 579.1701 | -1.20 | Flavonoids | 40581-17-7 |
| 4.06 | Quercetin | C_15_H_10_O_7_ | M+H | 303.0496 | -1.18 | Flavonoids | 117-39-5 |
| 0.63 | Trigonelline | C_7_H_7_NO_2_ | M+H | 138.055 | 0.52 | Alkaloids and derivatives | 535-83-1 |
| 4.00 | Cinnamic acid | C_9_H_8_O_2_ | M+H-H_2_O | 131.0492 | 0.71 | Phenolic acids and derivatives | 140-10-3;621-82-9;102-94-3 |
| 5.51 | Daidzein | C_15_H_10_O_4_ | M+H, M+Na | 255.0649 | -1.11 | Flavonoids | 486-66-8 |
| 3.78 | Isoferulic acid | C_10_H_10_O_4_ | M+H-H_2_O | 177.0546 | 0.09 | Phenolic acids and derivatives | 537-73-5;25522-33-2 |
| 3.34 | Sinapine | C_16_H_24_NO_5_^+^ | M+ | 310.1647 | -2.33 | Phenolic acids and derivatives | 84123-22-8;18696-26-9 |
| 7.82 | Chrysoeriol | C_16_H_12_O_6_ | M+H, M+Na | 301.0703 | -1.09 | Flavonoids | 491-71-4 |
| 6.14 | (+)-Syringaresinol | C_22_H_26_O_8_ | M+H-H_2_O, M+Na, M+H, M+, 2M+Na | 401.1591 | -0.98 | Lignans and derivatives | 21453-69-0 |
| 7.97 | Baicalein | C_15_H_10_O_5_ | M+H, 2M+K, M+Na | 271.0597 | -1.49 | Flavonoids | 491-67-8 |
| 5.37 | Baicalin | C_21_H_18_O_11_ | M+H, 2M+Na, M+Na | 447.0915 | -1.58 | Flavonoids | 21967-41-9 |
| 10.95 | Benzoic Acid | C_7_H_6_O_2_ | M+H | 123.0443 | 1.75 | Phenolic acids and derivatives | 100-52-7;99-10-5;55-21-0;65-85-0;303-07-1 |
| 13.67 | Nerolidol | C_15_H_26_O | M+NH4 | 240.232 | -0.82 | Terpenoids | 1119-38-6;7212-44-4;142-50-7 |
| 3.90 | Paeoniflorin | C_23_H_28_O_11_ | M+NH4, M+K, M+Na, 2M+Na, M+H-H_2_O | 498.1962 | -1.56 | - | 23180-57-6 |
| 5.01 | Albiflorin | C_23_H_28_O_11_ | M+H, M+NH4, 2M+NH4, 2M+Na, M+K | 481.1697 | -1.53 | - | 39011-90-0 |

**Table S2.** Identified compounds in TSPSS under negative ion mode.

| RT  (min) | Ingredient name | Formula | Adduct type | Observed  (m/z) | Error  (ppm) | Compounds | CAS No. |
| --- | --- | --- | --- | --- | --- | --- | --- |
| 7.20 | Medicoside E | C_47_H_76_O_18_ | M-H, M+FA-H | 973.4999 | -1.54 | Terpenoids | 169397-84-6 |
| 7.82 | Pinoquercetin | C_16_H_12_O_7_ | M-H_2_O-H | 297.0402 | -0.94 | Flavonoids | 491-49-6 |
| 7.82 | Kaempferide | C_16_H_12_O_6_ | M-H | 299.0558 | -1.18 | Flavonoids | 491-54-3 |
| 0.89 | Citric acid | C_6_H_8_O_7_ | M-H | 191.0187 | -5.17 | Organic acids and derivatives | 77-92-9 |
| 6.27 | Malic Acid | C_4_H_6_O_5_ | M-H | 133.013 | -9.52 | Organic acids and derivatives | 6915-15-7;97-67-6;617-48-1 |
| 0.97 | Uridine | C_9_H_12_N_2_O_6_ | M-H | 243.0617 | -2.39 | Nucleotides and derivatives | 58-96-8 |
| 14.78 | Succinic Acid | C_4_H_6_O_4_ | M-H | 117.0193 | -0.46 | Organic acids and derivatives | 110-15-6 |
| 8.62 | Formononetin | C_16_H_12_O_4_ | M-H, 2M-H | 267.0659 | -1.32 | Flavonoids | 485-72-3 |
| 4.74 | Hesperidin | C_28_H_34_O_15_ | M-H, M+Cl, M-H_2_O-H | 609.1818 | -1.15 | Flavonoids | 520-26-3 |
| 4.41 | Sinapic acid | C_11_H_12_O_5_ | M-H | 223.0605 | -3.02 | Phenolic acids and derivatives | 7361-90-2;530-59-6;7362-37-0; |
| 3.34 | Caffeic acid | C_9_H_8_O_4_ | M-H | 179.034 | -5.66 | Phenolic acids and derivatives | 331-39-5;4361-87-9;501-16-6 |
| 0.63 | Sucrose | C_12_H_22_O_11_ | M+Cl | 377.085 | -1.89 | Carbohydrates and derivatives | 57-50-1 |
| 7.46 | Vulgarin | C_15_H_20_O_4_ | M+FA-H | 309.1341 | -1.08 | Terpenoids | 3162-56-9 |
| 3.30 | Zizybeoside II | C_25_H_38_O_16_ | M-H, M+Cl, M+FA-H | 639.2138 | -0.71 | Carbohydrates and derivatives | 81417-79-0 |
| 4.24 | Scopoletin | C_10_H_8_O_4_ | M-H | 191.034 | -4.99 | Coumarins and derivatives | 92-61-5 |
| 5.17 | Lobetyolin | C_20_H_28_O_8_ | M-H, M+FA-H, M+Cl | 441.176 | -1.60 | volatile oil | - |
| 12.23 | Emodin | C_15_H_10_O_5_ | M-H | 269.0453 | -0.93 | Quinones | 518-82-1 |
| 4.64 | Azelaic Acid | C_9_H_16_O_4_ | M-H | 187.0966 | -5.17 | Lipids | 123-99-9 |
| 4.96 | Luteolin | C_15_H_10_O_6_ | M-H | 285.0402 | -0.93 | Flavonoids | 491-70-3 |
| 3.11 | Liquiritigenin | C_15_H_12_O_4_ | M+FA-H | 301.0714 | -1.23 | Flavonoids | 578-86-9 |
| 10.37 | Licoricesaponin B_2_ | C_42_H_64_O_15_ | M-H, M+Na-2H | 807.4166 | -0.77 | Terpenoids | 118536-86-0 |
| 11.24 | Licoricesaponin C2 | C_42_H_62_O_15_ | M-H, M+Na-2H | 805.4011 | -0.58 | Terpenoids | 118525-49-8 |
| 7.38 | Licoricesaponin A3 | C_48_H_72_O_21_ | M-H, M+Na-2H, M-2H | 983.4481 | -1.21 | Terpenoids | 118325-22-7 |
| 10.89 | Licoricesaponin J2 | C_42_H_64_O_16_ | M-H | 823.4102 | -2.44 | Terpenoids | 134250-13-8 |
| 4.72 | Umbelliferone | C_9_H_6_O_3_ | M-H | 161.0232 | -7.40 | Coumarins and derivatives | 93-35-6 |
| 5.17 | Kaempferol | C_15_H_10_O_6_ | M-H | 285.0402 | -1.07 | Flavonoids | 80714-53-0;520-18-3 |
| 4.49 | Naringin | C_27_H_32_O_14_ | M-H, M+FA-H, M+Cl | 579.1712 | -1.21 | Flavonoids | 10236-47-2 |
| 4.22 | Neoliquiritin | C_21_H_22_O_9_ | M-H, M+Cl, 2M-H | 417.1186 | -1.33 | Flavonoids | 5088-75-5 |
| 9.78 | Glycyrrhizin | C_42_H_62_O_16_ | M-H, M+K-2H, M+Na-2H | 821.3955 | -1.26 | Terpenoids | 1405-86-3;103000-77-7 |
| 5.21 | Neoisoliquiritin | C_21_H_22_O_9_ | M-H, M+Cl, 2M-H | 417.1185 | -1.36 | Flavonoids | 59122-93-9 |
| 5.21 | 7,4'-Dihydroxyflavone | C_15_H_10_O_4_ | M-H | 253.0502 | -1.70 | Flavonoids | 2196-14-7 |
| 7.53 | Gancaonin D | C_21_H_20_O_7_ | M+FA-H | 429.1185 | -1.60 | Flavonoids | 124596-88-9 |
| 10.42 | Pinocembrin | C_15_H_12_O_4_ | M-H | 255.0659 | -1.68 | Flavonoids | 480-39-7 |
| 10.44 | Prunetin | C_16_H_12_O_5_ | M-H | 283.0609 | -1.20 | Flavonoids | 552-59-0 |
| 12.52 | Glabridin | C_20_H_20_O_4_ | M-H | 323.1286 | -0.99 | Flavonoids | 59870-68-7 |
| 13.19 | Morusin | C_25_H_24_O_6_ | M-H | 419.1495 | -1.14 | Flavonoids | 62596-29-6 |
| 12.52 | 3-Hydroxyglabrol | C_25_H_28_O_5_ | M-H | 407.1861 | -0.73 | Flavonoids | 74148-41-7 |
| 4.04 | Protocatechuic acid | C_7_H_6_O_4_ | M-H | 153.0182 | -7.55 | Phenolic acids and derivatives | 99-50-3 |
| 6.47 | Isorhamnetin | C_16_H_12_O_7_ | M-H | 315.0509 | -0.48 | Flavonoids | 480-19-3 |
| 4.22 | Isoquercitrin | C_21_H_20_O_12_ | M-H | 463.088 | -0.44 | Flavonoids | 482-35-9;21637-25-2 |
| 2.93 | Coumarinic acid | C_9_H_8_O_3_ | M+FA-H | 209.0447 | -5.23 | Phenolic acids and derivatives | 495-79-4 |
| 8.73 | Dalbergin | C_16_H_12_O_4_ | M+FA-H | 313.0716 | -0.55 | Flavonoids | 482-83-7 |
| 3.79 | Taxifolin | C_15_H_12_O_7_ | M-H | 303.0508 | -0.86 | Flavonoids | 480-18-2 |
| 5.21 | Eriodictyol | C_15_H_12_O_6_ | M-H | 287.0558 | -1.15 | Flavonoids | 552-58-9 |
| 10.13 | Chrysin | C_15_H_10_O_4_ | M-H | 253.0502 | -1.65 | Flavonoids | 480-40-0 |
| 9.95 | Wogonin | C_16_H_12_O_5_ | M-H | 283.0608 | -1.30 | Flavonoids | 632-85-9 |
| 3.48 | (+)-Catechin | C_15_H_14_O_6_ | M-H | 289.0714 | -1.32 | Flavonoids | 154-23-4;490-46-0 |
| 8.33 | Santamarin | C_15_H_20_O_3_ | M+FA-H | 293.1392 | -0.97 | Terpenoids | 4290-13-5 |
| 9.27 | Wogonoside | C_22_H_20_O_11_ | M+H | 461.1096 | 3.86 | Flavonoids | 51059-44-0 |
| 4.26 | Acteoside | C_29_H_36_O_15_ | M-H, M+Na-2H | 623.1972 | -1.45 | Phenolic acids and derivatives | 61276-17-3 |

**Table S3.** Differential analysis of serum estradiol and progesterone levels.

| Items | Treatment | | | SEM | p-value | | |
| --- | --- | --- | --- | --- | --- | --- | --- |
|  | NC | GY | MY |  | Treatments | Time | Treatments  × Time |
| E2 | | | | | | | |
| Pre-pregnancy | 192.99±4.18Wx | 247.75±40.2w | 215.16±42.22W | 18.923 | 0.007 | <0.001 | 0.151 |
| Mid-pregnancy | 253.99±45.72bWw | 327.44±34.94av | 336.78±51.79aV |  |  |  |  |
| Late-pregnancy | 328.37±27.43V | 327.58±26.51v | 372.78±44.96V |  |  |  |  |
| P4 | | | | | | | |
| Pre-pregnancy | 125.66±  18.50VWw | 129.04±  13.23v | 123.84±  21.64w | 8.644 | 0.045 | <0.001 | 0.095 |
| Mid-pregnancy | 148.87±12.45Vv | 146.03±20.15v | 159.57±13.13v |  |  |  |  |
| Late-pregnancy | 113.96±8.31ABbWw | 105.46±21.38Bbw | 148.99±21.28Aavw |  |  |  |  |

Different letters in the same row indicate significant differences (^a,b,c^*p* <0.05, ^A,B,C^*p* <0.01 Different letters in the same column indicate significant differences (^v-z^*p* <0.05, ^V-Z^*p* < 0.01).

**Table S4.** Analysis of differences in foal body measurements and weight changes.

| Items | Treatment | | | SEM | p-value | | |
| --- | --- | --- | --- | --- | --- | --- | --- |
|  | NC | GY | MY |  | Treatments | Time | Treatments  × Time |
| Body weight | | | | | | | |
| 0d | 45.75±2.63bZ | 52.50±1.73aY | 48.75±4.19abY | 3.438 | <0.001 | <0.001 | 0.321 |
| 30d | 86.25±1.71Y | 91.75±5.36X | 92.00±6.27X |  |  |  |  |
| 60d | 108.25±5.38X | 116.75±9.14W | 119.00±6.63W |  |  |  |  |
| 90d | 123.00±4.97bW | 142.50±12.15aV | 140.00±12.78aV |  |  |  |  |
| 120d | 139.25±4.65V | 156±19.51V |  |  |  |  |  |
| Body height | | | | | | | |
| 0d | 90.75±3.69Yx | 97.75±6.45Yy | 93.25±3.20Xy | 2.028 | <0.001 | <0.001 | 0.666 |
| 30d | 102.50±6.35bXw | 108.50±2.38abXx | 110.25±2.06aWx |  |  |  |  |
| 60d | 110.00±4.16bWXw | 118.75±4.11aWv | 116.00±1.42aVw |  |  |  |  |
| 90d | 118.50±5.97bVWv | 125.50±3.11aVWv | 120.50±1.29abVv |  |  |  |  |
| 120d | 123±4.76Vv | 128±2.94Vv |  |  |  |  |  |
| Body oblique length | | | | | | | |
| 0d | 76.00±5.35Xy | 78.00±2.16Xy | 76.50±6.25Xy | 1.94 | 0.066 | <0.001 | 0.986 |
| 30d | 85.50±3.70Wx | 89.50±4.93Wx | 88.50±3.70Wx |  |  |  |  |
| 60d | 92.75±3.30Ww | 97.00±4.24VWw | 96.25±1.71VWw |  |  |  |  |
| 90d | 102.50±1.92Vv | 104.00±3.16Vv | 105.00±5.23Vv |  |  |  |  |
| 120d | 107.25±1.71Vv | 109.50±3.32Vv |  |  |  |  |  |
| Chest circumference | | | | | | | |
| 0d | 84.25±2.50bXy | 88.75±1.71aY | 87.00±2.16abXy | 2.035 | 0.004 | <0.001 | 0.989 |
| 30d | 102.50±3.11Wx | 105.25±2.63X | 106.25±4.27Wx |  |  |  |  |
| 60d | 110.25±4.99VWw | 114.75±4.03W | 115.75±2.22Vw |  |  |  |  |
| 90d | 119.25±6.70Vv | 123.00±4.08V | 122.00±4.55Vv |  |  |  |  |
| 120d | 121±6.16Vv | 126.25±4.11V |  |  |  |  |  |
| Cannon circumference | | | | | | | |
| 0d | 13.00±0.00Wx | 13.25±0.50Xy | 12.75±0.50Wx | 0.241 | 0.012 | <0.001 | 0.553 |
| 30d | 14.25±0.50Vw | 14.75±0.50Wx | 14.25±0.50Vw |  |  |  |  |
| 60d | 14.50±0.58Vw | 15.00±0.00VWwx | 14.75±0.50Vvw |  |  |  |  |
| 90d | 14.75±0.50bVw | 15.75±0.50aVv | 15.25±0.50abVv |  |  |  |  |
| 120d | 15.50±0.58Vv | 15.50±0.58Vvw |  |  |  |  |  |

Different letters in the same row indicate significant differences (^a,b,c^*p* <0.05, ^A,B,C^*p* <0.01 Different letters in the same column indicate significant differences (^v-z^*p* <0.05, ^V-Z^*p* < 0.01).

**Table S5.** Differential analysis of serum cytokines, immunological markers, antioxidant indicators, and growth hormone in foals.

| Items | Treatment | | | SEM | p-value | | |
| --- | --- | --- | --- | --- | --- | --- | --- |
|  | NC | GY | MY |  | Treatments | Time | Treatments  × Time |
| IL-6 | | | | | | | |
| 0d | 389.49±52.33bXx | 488.68±20.97aWy | 482.56±53.67avw | 18.661 | 0.055 | <0.001 | <0.001 |
| FQ | 516.85±41.06aVWw | 438.06±30.15Xz | 448.68±36.55bw |  |  |  |  |
| 30d | 522.16±29.79bVvWw | 589.11±15.85aVv | 517.67±58.45bvw |  |  |  |  |
| 60d | 446.64±36.70bWXx | 552.37±28.79aVwx | 509.50±42.89avw |  |  |  |  |
| 90d | 568.70±20.97Vvw | 580.53±20.85Vvw | 544.61±37.87v |  |  |  |  |
| 120d | 583.39±52.19Vv | 529.09±9.33Vx |  |  |  |  |  |
| IL-10 | | | | | | | |
| 0d | 70.76±5.07bv | 82.11±7.64aVv | 77.29±4.29abVv | 3.126 | 0.032 | <0.001 | 0.548 |
| FQ | 57.9225±5.06341w | 58.855±5.14564wX | 64.26±2.24483Ww |  |  |  |  |
| 30d | 65.16±8.41vw | 66.21±9.21WwX | 61.81±7.38Ww |  |  |  |  |
| 60d | 65.23±7.80vw | 69.04±4.09VWwX | 65.59±6.72VWw |  |  |  |  |
| 90d | 71.92±5.73v | 80.13±5.83VvW | 76.63±5.24Vv |  |  |  |  |
| 120d | 68.11±4.78v | 71.99±7.41VWwX |  |  |  |  |  |
| IFN-γ | | | | | | | |
| 0d | 163.25±11.11bWwx | 183.92±15.62aWx | 185.66±6.46aWwXx | 8.353 | <0.001 | <0.001 | 0.004 |
| FQ | 197.96±15.99Vv | 191.51±7.67VWwx | 177.55±15.52Xx |  |  |  |  |
| 30d | 162.03±17.11BWwx | 221.77±21.12AVv | 212.61±15.61AVvW |  |  |  |  |
| 60d | 191.59±11.13bVvw | 221.77±15.86aVv | 222.12±13.27aVv |  |  |  |  |
| 90d | 157.41±7.64BWx | 203.98±15.26AVvWwx | 202.76±8.91AVvWwX |  |  |  |  |
| 120d | 186.10±37.32Vvwx | 216.88±21.98vw |  |  |  |  |  |
| TNF-α | | | | | | | |
| 0d | 149.62±15.79bw | 177.39±15.53aVv | 166.94±9.03ab | 6.542 | <0.001 | 0.004 | 0.071 |
| FQ | 156.49±12.35vw | 153.82±10.51Ww | 156.76±13.90 |  |  |  |  |
| 30d | 160.87±4.58Bvw | 189.70±13.19AVv | 168.01±8.62AB |  |  |  |  |
| 60d | 149.89±15.03Bbw | 189.70±9.39AaVv | 173.28±7.35AaB |  |  |  |  |
| 90d | 151.94±19.92w | 168.01±9.97VvWw | 156.59±12.48 |  |  |  |  |
| 120d | 174.26±8.59v | 176.86±22.79vW |  |  |  |  |  |
| SOD | | | | | | | |
| 0d | 9.36±0.97B | 13.37±1.36A | 13.31±1.74Av | 0.769 | <0.001 | 0.395 | 0.114 |
| FQ | 10.24±2.25 | 12.04±1.16 | 11.25±1.22vw |  |  |  |  |
| 30d | 11.07±1.75 | 12.42±1.01 | 11.10±2.17vw |  |  |  |  |
| 60d | 11.26±1.05ABb | 13.20±0.86Aa | 10.51±0.86Bbw |  |  |  |  |
| 90d | 9.35±1.58 | 11.68±2.21 | 10.98±1.51vw |  |  |  |  |
| 120d | 11.24±1.83 | 11.34±1.44 |  |  |  |  |  |
| MDA | | | | | | | |
| 0d | 0.71±0.09VWwx | 0.56±0.14Vvwx | 0.58±0.15 | 0.066 | <0.001 | 0.001 | 0.263 |
| FQ | 0.97±0.26Vv | 0.67±0.11Vv | 0.68±0.14 |  |  |  |  |
| 30d | 0.90±0.11aVvWw | 0.57±0.18bVvw | 0.57±0.19 |  |  |  |  |
| 60d | 0.78±0.06AVvWwx | 0.38±0.03CVx | 0.57±0.07B |  |  |  |  |
| 90d | 0.66±0.09Wx | 0.48±0.10Vwx | 0.49±0.13 |  |  |  |  |
| 120d | 0.66±0.12Wx | 0.61±0.08Vvw |  |  |  |  |  |
| T-AOC | | | | | | | |
| 0d | 11.45±0.46bVv | 13.22±1.22av | 11.43±1.04bVv | 0.512 | <0.001 | <0.001 | 0.629 |
| FQ | 9.75±1.04VWwX | 11.77±1.69vw | 10.93±1.02VvWw |  |  |  |  |
| 30d | 9.91±0.76VWw | 11.05±1.30wx | 10.32±1.48VvWw |  |  |  |  |
| 60d | 8.51±0.38bXxY | 10.46±1.03awxy | 9.21±1.04abVWwx |  |  |  |  |
| 90d | 9.01±0.34VWwXxY | 9.33±1.40xy | 8.55±0.85Wx |  |  |  |  |
| 120d | 8.20±0.56xY | 8.69±0.52y |  |  |  |  |  |
| IgA | | | | | | | |
| 0d | 2.64±0.44bVv | 3.90±0.24aVv | 2.90±0.46bV | 0.172 | <0.001 | <0.001 | 0.01 |
| FQ | 1.39±0.49bwX | 2.34±0.31aWwx | 2.18±0.18aW |  |  |  |  |
| 30d | 1.72±0.33WwX | 2.03±0.53Wx | 2.08±0.44W |  |  |  |  |
| 60d | 1.49±0.23bwX | 2.72±0.26aWw | 1.87±0.29bW |  |  |  |  |
| 90d | 1.80±0.17abWwX | 2.11±0.27aWx | 1.66±0.26bW |  |  |  |  |
| 120d | 2.41±0.40VvW | 2.41±0.24Wwx |  |  |  |  |  |
| IgG | | | | | | | |
| 0d | 22.06±1.86BVv | 26.55±1.37AVv | 22.36±1.67BV | 0.950 | <0.001 | <0.001 | 0.025 |
| FQ | 12.69±2.037Ww | 15.64±2.10VWXx | 14.15±1.84W |  |  |  |  |
| 30d | 12.48±2.83Ww | 14.18±2.17Xx | 14.03±1.95W |  |  |  |  |
| 60d | 12.54±1.55BWw | 19.61±2.1AVw | 14.52±1.40BW |  |  |  |  |
| 90d | 13.22±1.82vWw | 15.15±1.53Xx | 15.21±1.29W |  |  |  |  |
| 120d | 16.22±2.32vW | 16.47±1.81VWXx |  |  |  |  |  |
| IgM | | | | | | | |
| 0d | 1.52±0.19bV | 1.88±0.16aVv | 1.55±0.26abV | 0.106 | 0.026 | <0.001 | 0.073 |
| FQ | 0.96±0.15abW | 0.86±0.19bYy | 1.21±0.19aW |  |  |  |  |
| 30d | 0.89±0.28W | 0.98±0.20Yy | 1.00±0.13X |  |  |  |  |
| 60d | 1.00±0.12bW | 1.42±0.17aVWw | 1.06±0.30bX |  |  |  |  |
| 90d | 0.94±0.12W | 1.11±0.11VWXxYy | 1.14±0.13WX |  |  |  |  |
| 120d | 1.25±0.39VW | 1.33±0.62VWwXx |  |  |  |  |  |
| GH | | | | | | | |
| 0d | 5.84±0.67BcXx | 11.62±0.84AaVWw | 9.77±1.09AbVWw | 0.470 | <0.001 | <0.001 | <0.001 |
| FQ | 7.14±1.05BWwXx | 10.04±0.83AWwx | 11.33±11.33AVv |  |  |  |  |
| 30d | 9.84±1.49AVv | 6.24±0.90BXy | 6.03±0.54BXx |  |  |  |  |
| 60d | 7.53±0.87BbWwX | 10.69±0.87AaWx | 8.80±1.27ABbWw |  |  |  |  |
| 90dc | 6.94±0.86BcwXx | 11.74±1.24AaVWw | 8.75±0.70BbWw |  |  |  |  |
| 120d | 9.19±0.94BVvW | 13.38±0.79AVv |  |  |  |  |  |
| IGF-1 | | | | | | | |
| 0d | 218.10±12.31BbWXx | 282.94±9.03AaVvwx | 246.93±9.35ABbVvW | 11.359 | <0.001 | <0.001 | <0.001 |
| FQ | 252.67±19.09AaBWw | 260.53±35.49AaVWxy | 191.43±25.79BbWw |  |  |  |  |
| 30d | 188.08±7.96bXy | 225.46±10.21aXy | 229.58±14.71aVvWw |  |  |  |  |
| 60d | 188.51±5.94BbXy | 303.36±18.22AaVvw | 264.75±15.13AaVv |  |  |  |  |
| 90d | 229.84±5.09BbWwx | 271.67±5.95AaVWwx | 236.67±10.75ABbVvW |  |  |  |  |
| 120d | 291±21.72bVv | 320.76±9.51aVv |  |  |  |  |  |
| T3 | | | | | | | |
| 0d | 0.56±0.04B | 0.69±0.02Av | 0.59±0.03BxW | 0.017 | <0.001 | 0.286 | 0.002 |
| FQ | 0.58±0.02Bb | 0.63±0.06ABbvw | 0.69±0.02AaVv |  |  |  |  |
| 30d | 0.60±0.04Bb | 0.68±0.01Aav | 0.66±0.04ABaVvWw |  |  |  |  |
| 60d | 0.57±0.03C | 0.69±0.02Av | 0.63±0.02BVWwx |  |  |  |  |
| 90d | 0.58±0.03b | 0.66±0.04avw | 0.64±0.05abVvWwx |  |  |  |  |
| 120d | 0.61±0.04 | 0.62±0.03w |  |  |  |  |  |
| T4 | | | | | | | |
| 0d | 15.91±1.05BWwx | 21.14±1.23AV | 20.412±1.83AVvWw | 0.794 | <0.001 | <0.001 | 0.005 |
| FQ | 19.16±1.69bVv | 19.98±2.03abV | 22.30±1.63aVv |  |  |  |  |
| 30d | 16.00±1.19BbWwx | 19.93±1.10AaV | 18.29±1.75ABaVWwx |  |  |  |  |
| 60d | 16.64±1.38Vw | 19.74±1.48V | 19.18±2.70VWwx |  |  |  |  |
| 90d | 15.66±1.10BWwx | 20.88±1.78AV | 16.48±1.09BWx |  |  |  |  |
| 120d | 14.41±1.61Wx | 15.49±1.39W |  |  |  |  |  |
| INS | | | | | | | |
| 0d | 13.44±0.66B | 15.89±0.68A | 15.54±1.10Av | 0.468 | <0.001 | 0.124 | 0.091 |
| FQ | 13.22±0.62B | 15.16±1.04A | 15.29±0.45Av |  |  |  |  |
| 30d | 13.50±1.34 | 14.91±0.72 | 13.12±1.45w |  |  |  |  |
| 60d | 13.64±0.47b | 15.08±1.16a | 14.40±0.76abvw |  |  |  |  |
| 90d | 13.35±0.77B | 16.09±0.81A | 14.38±1.50ABvw |  |  |  |  |
| 120d | 14.18±0.27 | 14.87±0.98 |  |  |  |  |  |
| INS | | | | | | | |
| 0d | 13.44±0.66B | 15.89±0.68A | 15.54±1.10Av | 0.468 | <0.001 | 0.124 | 0.091 |
| FQ | 13.22±0.62B | 15.16±1.04A | 15.29±0.45Av |  |  |  |  |
| 30d | 13.50±1.34 | 14.91±0.72 | 13.12±1.45w |  |  |  |  |
| 60d | 13.64±0.47b | 15.08±1.16a | 14.40±0.76abvw |  |  |  |  |
| 90d | 13.35±0.77B | 16.09±0.81A | 14.38±1.50ABvw |  |  |  |  |
| 120d | 14.18±0.27 | 14.87±0.98 |  |  |  |  |  |

Different letters in the same row indicate significant differences (^a,b,c^*p* <0.05, ^A,B,C^*p* <0.01 Different letters in the same column indicate significant differences (^v-z^*p* <0.05, ^V-Z^*p* < 0.01).

**Table S6.** Differential analysis of short-chain fatty acids concentrations in Foal Feces.

| Items | Treatment | | | SEM | p-value | | |
| --- | --- | --- | --- | --- | --- | --- | --- |
|  | NC | GY | MY |  | Treatments | Time | Treatments  × Time |
| Acetic acid | | | | | | | |
| 0d | 112.69±17.63Ww | 118.37±26.13W | 120.95±19.72vw | 7.287 | 0.967 | <0.001 | 0.915 |
| FQ | 116.08±19.18Ww | 113.46±18.81W | 112.95±18.53vw |  |  |  |  |
| 30d | 120.6±8.94aWw | 113.48±6.00abW | 108.63±4.92bw |  |  |  |  |
| 60d | 124.16±14.34VWw | 123.39±10.82W | 116.7±10.84vw |  |  |  |  |
| 90d | 126.74±15.84VWw | 123.52±12.00W | 133.78±7.39v |  |  |  |  |
| 120d | 155.44±10.02Vv | 158.37±5.61V |  |  |  |  |  |
| Propionic acid | | | | | | | |
| 0d | 32.14±8.58VWw | 31.81±6.39W | 32.17±9.31 | 2.595 | 0.687 | <0.001 | 0.924 |
| FQ | 29.41±5.25Ww | 28.42±5.36W | 27.88±5.15 |  |  |  |  |
| 30d | 30.28±4.57Ww | 29.88±5.02W | 29±5.31 |  |  |  |  |
| 60d | 35.75±2.98aVvWw | 31.77±4.19abW | 29.05±1.65b |  |  |  |  |
| 90d | 35.62±4.81VvWw | 34.83±3.57W | 36.41±3.12 |  |  |  |  |
| 120d | 43.43±4.38Vv | 46.22±2.34V |  |  |  |  |  |
| Isobutyric acid | | | | | | | |
| 0d | 1.15±0.35Ww | 1.17±0.12Ww | 1.38±0.16VW | 0.139 | 0.692 | <0.001 | 0.577 |
| FQ | 1.27±0.25Ww | 1.32±0.26Ww | 1.65±0.23V |  |  |  |  |
| 30d | 1.26±0.16Ww | 1.21±0.18Ww | 1.19±0.13W |  |  |  |  |
| 60d | 1.37±0.18Ww | 1.48±0.29VWw | 1.23±0.17W |  |  |  |  |
| 90d | 1.46±0.36Vw | 1.32±0.29Ww | 1.39±0.23VW |  |  |  |  |
| 120d | 2.04±0.41Vv | 2.05±0.55Vv |  |  |  |  |  |
| N-butyric acid | | | | | | | |
| 0d | 14.46±2.98Ww | 15.97±2.90W | 13.72±2.96V | 1.545 | 0.687 | <0.001 | 0.009 |
| FQ | 5.07±0.88AaXy | 3.54±0.67ABbY | 2.59±0.69BbWx |  |  |  |  |
| 30d | 8.12±2.36bWXxy | 7.79±1.81bXY | 13.15±2.98aVv |  |  |  |  |
| 60d | 13.78±2.85AaWw | 12.01±2.17AaBWX | 7.24±1.94BbWw |  |  |  |  |
| 90d | 12.82±1.94Wwx | 15.82±3.64W | 17.03±3.21Vv |  |  |  |  |
| 120d | 33.23±6.29Vv | 36.84±5.74V |  |  |  |  |  |
| Isovaleric acid | | | | | | | |
| 0d | 1.91±0.23XxY | 1.91±0.33Xy | 2.33±0.32WwX | 0.250 | 0.196 | <0.001 | 0.002 |
| FQ | 1.82±0.26xY | 1.64±0.27Xy | 1.57±0.17Xx |  |  |  |  |
| 30d | 2.51±0.35abWwXxY | 2.44±0.65bWXxy | 3.38±0.59aVv |  |  |  |  |
| 60d | 3.21±0.77abVWw | 3.64±0.61aWw | 2.34±0.46bWwX |  |  |  |  |
| 90d | 2.91±0.52WwX | 3.26±0.72Wwx | 3.14±0.29VvW |  |  |  |  |
| 120d | 4.11±0.50Vv | 5.16±0.74Vv |  |  |  |  |  |
| N-pentanoic acid | | | | | | | |
| 0d | 1.42±0.32WwXx | 1.48±0.36WwX | 1.63±0.26WwX | 0.185 | 0.208 | <0.001 | 0.083 |
| FQ | 1.02±0.11Xx | 1.16±0.18Xx | 1.13±0.15Xx |  |  |  |  |
| 30d | 1.61±0.24WwX | 1.4±0.28WX | 1.69±0.26VvWw |  |  |  |  |
| 60d | 1.77±0.40WwX | 1.71±0.26WwXx | 1.56±0.47WX |  |  |  |  |
| 90d | 2.12±0.45Ww | 2.17±0.34Ww | 2.35±0.38Vv |  |  |  |  |
| 120d | 2.90±0.30Vv | 3.00±0.29Vv |  |  |  |  |  |

Different letters in the same row indicate significant differences (^a,b,c^*p* <0.05, ^A,B,C^*p* <0.01 Different letters in the same column indicate significant differences (^v-z^*p* <0.05, ^V-Z^*p* < 0.01).
