# Supplementary material for: Maternal Taishan Panshi San supplementation is associated with improved foal growth performance, accompanied by alterations in gut microbiota and metabolic profiles
Source: Front Microbiol. 2026 Apr 20;17:1787938. doi: 10.3389/fmicb.2026.1787938 (PMC13136102; doi:10.3389/fmicb.2026.1787938)
Supplement: Supplementary file 2 [file Table_2.pdf]

# Appendix

The primer sequences of RT-qPCR:

| Gene Name | Sequences (5′～3′) | Production length (bp) | Genbank accession NO. |
|---|---|---|---|
| *SLC2A1-F* | CCATTGCTGTCTCTGGCTTC | 184 | NM_001163971.2 |
| *SLC2A1-R* | GCAATCTCATCGAAGGTCCG | | |
| *SLC6A4-F* | CAACTGTTACCAAGACGCCC | 133 | NM_001081824.2 |
| *SLC6A4-R* | CTCAGCCATATACCCCAGCA | | |
| *SLC7A1-F* | TTGTGGGCTTTGACTGCATC | 249 | XM_023621462.1 |
| *SLC7A1-R* | TGGAAAGAGCACAGAGGGAG | | |
| *SLC27A1-F* | TCAGGTGATGTGCTGGTGAT | 187 | XM_023625410.1 |
| *SLC27A1-R* | CTGCTTTGCCCTCTACTCCT | | |
| *SLC38A1-F* | GTGACCTGCCTACTCTTGGT | 222 | XM_014740928.2 |
| *SLC38A1-R* | GCTGACCAGGGAAAACAACA | | |
| *SLC38A2-F* | TGCCAATGAAGGAGGGTCTT | 245 | XM_001489523.6 |
| *SLC38A2-R* | GGATGAGCACCAATGACACC | | |
| *SLC38A4-F* | ACAGGGGTGCTTGTCATGTA | 217 | XM_023643434.1 |
| *SLC38A4-R* | GAACAGGACAATGGGCACAG | | |
| *CD36-F* | CTCTGAGAACCACACCGTCT | 243 | XM_005609038.3 |
| *CD36-R* | AAGAACGGGTCCTTGTAGCC | | |
| *VEGFA-F* | AACCTCACCAAAGCCAACAC | 211 | NM_001081821.1 |
| *VEGFA-R* | TTTAACTCAAGCTGCCTCGC | | |
| *NOS3-F* | GTTTGTCTGCGGTGATGTCA | 156 | XM_001504650.6 |
| *NOS3-R* | GTGAGCCCGAAAATGTCCT | | |
| *IGF-2-F* | GTACAACGCCTGGAAGCAAT | 190 | NM_001114539.2 |
| *IGF-2-R* | GCTGGACACCTCGACAAAG | | |
| *AKT-F* | AGGCGAACTGGATGTGAAGA | 196 | XM_023636992.1 |
| *AKT-R* | GCACGCCTTAACACTGTCAA | | |
| *HTR2A-5-F* | CTCGTCATCATGGCAGTGTC | 198 | NM_001081784.1 |

| | | | |
|---|---|---|---|
| *HTR2A-5-R* | GGAGAAGAGCACATCCAGGT | | |
| *HTR7-F* | GGGAAATGCATGGCGAAGAT | 151 | XM_023642870.1 |
| *HTR7-R* | CTGCGGTGGAGTAAATCGTG | | |
| *DNMT3B-F* | CCCATTCGAGTCTTGTCCCT | 229 | XM_023626332.1 |
| *DNMT3B-R* | GGCTTCCACCAATCACCAAG | | |
